# Risk-stratification machine learning model using demographic factors, gynaecological symptoms and β-catenin for endometrial hyperplasia and carcinoma: a cross-sectional study

**DOI:** 10.1186/s12905-023-02790-6

**Published:** 2023-11-27

**Authors:** Rina Masadah, Aries Maulana, Berti Julian Nelwan, Mahmud Ghaznawie, Upik Anderiani Miskad, Suryani Tawali, Syahrul Rauf, Bumi Herman

**Affiliations:** 1 Department of Pathology Anatomy, Faculty of Medicine, Hasanuddin University, Makassar, Indonesia; 2 Department of Family Medicine and Preventive Medicine, Faculty of Medicine, Hasanuddin University, Makassar, Indonesia; 3 Departement of Obstetric and Gynecology, Faculty of Medicine, Hasanuddin University, Makassar, Indonesia; 4 College of Public Health Science, Chulalongkorn University, Bangkok, Thailand

**Keywords:** β-catenin, Endometrial hyperplasia, Endometrial carcinoma, Gynecological symptoms, Immunohistochemistry staining, Risk-stratification, Neural network, Decision Tree

## Abstract

**Background:**

Demographic features, suggestive gynaecological symptoms, and immunohistochemical expression of endometrial β-catenin have a prognostic capacity for endometrial hyperplasia and carcinoma. This study assessed the interaction of all variables and developed risk stratification for endometrial hyperplasia and carcinoma.

**Methods:**

This cross-sectional study was conducted from January 2023 to July 2023 at two teaching hospitals in Makassar Indonesia. Patients (< 70 years old) with suggestive symptoms of endometrial hyperplasia or carcinoma or being referred with disease code N.85 who underwent curettage and/or surgery for pathology assessment except those receiving radiotherapy, or chemotherapy, presence of another carcinoma, coagulation disorder, and history of anti-inflammatory drug use and unreadable samples. Demographic, and clinical symptoms were collected from medical records. Immunohistochemistry staining using mouse-monoclonal antibodies determined the β-catenin expression (percentage, intensity, and H-score) in endometrial tissues. Ordinal and Binary Logistic regression identified the potential predictors to be included in neural networks and decision tree models of histopathological grading according to the World Health Organization/WHO grading classification.

**Results:**

Abdominal enlargement was associated with worse pathological grading (adjusted odds ratio/aOR 6.7 95% CI 1.8–24.8). Increasing age (aOR 1.1 95% CI 1.03–1.2) and uterus bleeding (aOR 5.3 95% CI 1.3–21.6) were associated with carcinoma but not with %β-catenin and H-Score. However, adjusted by vaginal bleeding and body mass index, lower %β-catenin (aOR 1.03 95% 1.01–1.05) was associated with non-atypical hyperplasia, as well as H-Score (aOR 1.01 95% CI 1.01–1.02). Neural networks and Decision tree risk stratification showed a sensitivity of 80-94.8% and a specificity of 40.6–60% in differentiating non-atypical from atypical and carcinoma. A cutoff of 55% β-catenin area and H-Score of 110, along with other predictors could distinguish non-atypical samples from atypical and carcinoma.

**Conclusion:**

Risk stratification based on demographics, clinical symptoms, and β-catenin possesses a good performance in differentiating non-atypical hyperplasia with later stages.

**Supplementary Information:**

The online version contains supplementary material available at 10.1186/s12905-023-02790-6.

## Introduction

### Epidemiology

Endometrial hyperplasia is characterized by an increase of gland proportions to stroma in endometrial tissue, which in certain conditions may lead to endometrial cancer. Stratification of endometrial hyperplasia was based on the presence of atypical nuclear [1] where the atypical form is associated with a higher risk of cancer progression. In a nested-cohort study, approximately 6 months diagnosed with endometrial hyperplasia, around 1.73% of participants developed carcinoma but the 20-year progression risk was lower in non-atypical individuals compared to atypical (5% versus 28%) [2]. Later stages (including atypical hyperplasia and carcinoma) require aggressive treatment, such as hysterectomy [3], thus creating a problem with those who prefer to preserve fertility. Simple risk stratification is needed to screen the patient and efficiently allocate and maximize the performance of the required examination (including biopsy for pathological examination) as a study in Korea demonstrated a progression to endometrial carcinoma among women with endometrial hyperplasia who underwent repeated biopsies [4]. A good risk stratification should possess a better diagnostic performance to reduce the unnecessary burden of diagnostic and improper treatment. Risk stratification may include demographic and clinical symptoms associated with endometrial cancer, as well as blood biomarker and immunohistochemistry staining.

Age, obesity, and certain chronic diseases such as diabetes and hypertension were associated with endometrial cancer and hyperplasia, particularly among Hispanic women [5] and Chinese women [6]. Moreover, parity was associated with a lower risk of endometrial cancer [7]. A meta-analysis demonstrates the diagnostic ability of gynaecological symptoms to differentiate uterine cancer. The sensitivity and specificity of using these symptoms were lower than 70%, except for postmenopausal bleeding with sensitivity ranging from 67 to 93% and specificity of 63–84% [8].

Immunohistochemistry staining of β-catenin in endometrial tissue also possesses a potential prognostic capacity. Along with E-Cadherin, β-catenin, an epithelial cell adhesion molecule, has a crucial role in the Wnt signal transduction pathway, affecting the epithelial integrity [9]. Wnt-signalling activation enables β-catenin to bypass the inhibitory control of a cytoplasmic destruction complex, facilitating the translocation of β-catenin into the nucleus and subsequent activation of Wnt target genes [10]. Abnormal expression of β-catenin in cells also affects migration and cell invasion, and it arises from the disruption of the gene associated with β-catenin (specifically Catenin Beta-1/CTNNB-1) which plays a significant role in endometrial carcinoma progression [11] Moreover, nuclear expression of β-catenin was more frequent in endometrioid adenocarcinoma [12]. A murine-based study revealed the impact of deletion of exon 3 of CTNNB1 on endometrial hyperplasia [13]. Further study identified β-catenin capacity as an immunohistochemical surrogate of CTNNB1 exon 3 mutations, showing that β-catenin expression, particularly the nuclear expression possesses a good prognostic factor for endometrial carcinoma and may reflect the mutation of CTNNB1 gene [14].

We intended to assess interactions between associated factors of endometrial hyperplasia and carcinoma, the β-catenin expression in endometrial tissue, and the level of pathology in endometrial tissue. Moreover, we developed a novel risk-stratification system combining demography, clinical symptoms and β-catenin expression to classify the pathology level of endometrial hyperplasia and carcinoma among patients with gynaecological symptoms.

## Methodology

### Study design and target population

This is a cross-sectional study conducted between January 2023 and June 2023 at two teaching hospitals in Makassar Indonesia. Any patients with high suspicion of endometrial hyperplasia and/or endometrioid endometrial carcinoma (International Classification of Disease/ICD-10 code N.85) referred for pathology assessment after curettage and/or surgical biopsy or hysterectomy were included, except for those above 70 years old, receiving radiotherapy, or chemotherapy, presence of another carcinoma, coagulation disorder, and history of anti-inflammatory drug use. Moreover, samples with the presence of dominant hemorrhagic or illegible for tissue processing were excluded, unless successful re-sampling had been performed.

### Variables and tools

We obtained demographic data from medical records, including age at diagnosis, body mass index, number of parity, abortion and miscarriage, list of clinical symptoms and referral diagnosis by the gynaecologist. These data were collected during the initial admission of the patient at the hospital.

### Preparation of sample

We obtained the samples from either a curettage or a surgical procedure (hysterectomy). Block and section preparation was based on guideline [15] with some modifications. Fixation of each specimen block was applied using formalin and embedded with paraffin. This block was then resected with microtome with the size of 3 μm then incubated in a water bath at 60 degrees Celsius, and placed on the poly-l-silane slides. Before staining, these slides were immersed in Xylol solution for five minutes, followed by 95% alcohol for two minutes, and 70% alcohol for two minutes before rinsing with water. The first staining involved immersion of slides into Hematoxylin Mayer solution for 15 min, and Eosin 1% for 5 min after the slides had been rinsed with water between two staining sessions. These slides were then dehydrated using graded alcohol solution levels (70% and 95%) for 2–5 min each followed by carbol xylol for five minutes and covered with glass.

### Immunohistochemistry staining

This study modified a procedure from one study [16] as the cited study combined the staining of both β-catenin and CD10 (Cluster of Differentiation 10). Immunohistochemical staining began with deparaffinization of samples with xylene for five minutes two times and rehydration with graded alcohol solutions (96%, 80%, and 70%) for five minutes for each solution. These samples were then soaked into a Tris Buffer Saline (TBS) solution and heated using a microwave for 10 min followed by a cooling down process and washed using Phosphate Buffer Saline (PBS) two times for 5 min. The edge of the tissue was marked and these samples underwent a peroxide block for 15 min, and a protein block for five minutes (with PBS rinsing between these steps).

A β-Catenin mouse-monoclonal antibody (Cell Marque© The Netherlands) was given for 10 min followed by a PBS rinse twice, each for five minutes. This was then followed by HRP (Horseradish Peroxidase) (Cell Marque© The Netherlands) and rinsing with PBS twice, each for five minutes. Furthermore, the preparations were incubated with chromogen Diaminobenzidine (DAB) and washed with running water for 5 min, then immersed in hematoxylin solution for 5 min. The preparations were then washed again with running water. Subsequently, dehydration was carried out with graded alcohol (70% alcohol, 80% alcohol, 96% alcohol) for 5 min each and then cleared with two Xylol solutions for 5 min each. The slides were dried and then covered with deck glass.

### Interpretation of immunohistochemical staining results

Positive expression of β-catenin will appear brown on the nuclear, membrane and/or cytoplasm of tumour cells and the nuclear, membrane and/or cytoplasm of endometrial glands with hyperplasia. All slides that had been stained with the immunohistochemical method were assessed by two gynaecological pathologists independently.

### Scoring technique

**Fig. 1:**
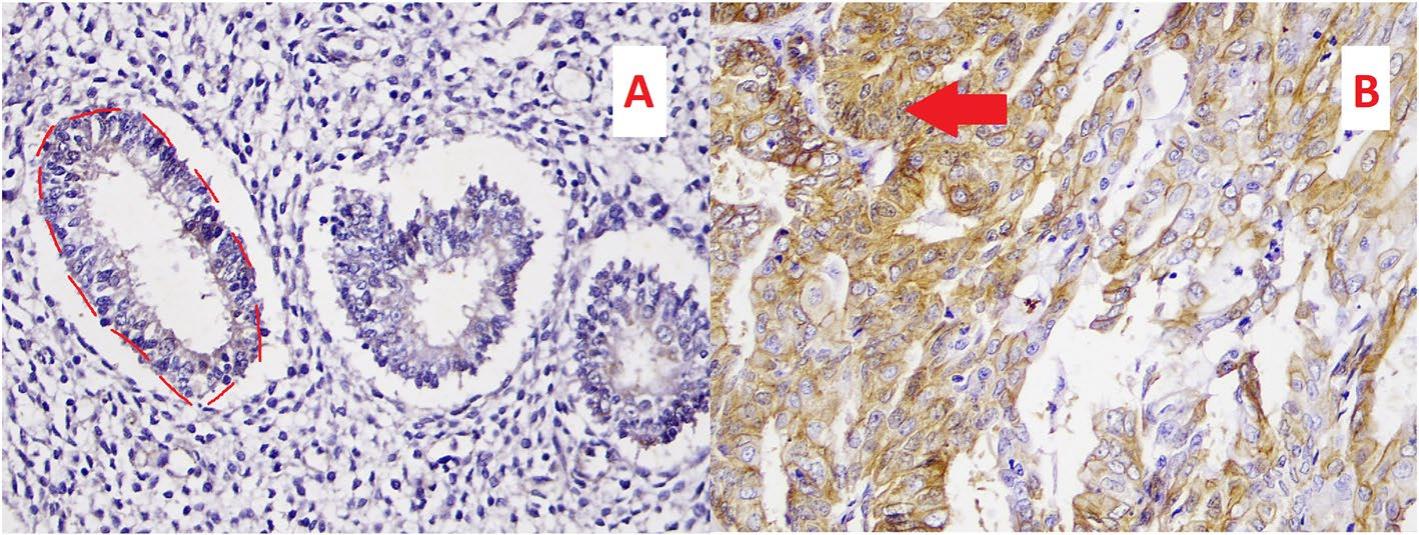
Expression of β-catenin in non-atypical hyperplasia and carcinoma. The non-atypical hyperplasia appears bluish with increased stromal cells and intact gland, marked with a red-dash line (**A**). Whereas expression of β-catenin, shown by red arrows, with irregular cell borders and poor differentiation, suggesting carcinoma (**B**)

Expression of β-catenin protein binding in endometrial tissue with hyperplasia, both atypical and non-atypical could be seen as a brownish chromogen substance on the cell membrane and/or cytoplasm, which was observed with a light microscope at 10 high-power fields with 400x magnification. Intensity score ranged from 0 to 3, whereas percentage points ranged from 0 to 100%. The H-score was defined as the multiplication of percentage and intensity score. The classification of endometrial hyperplasia was following the World Health Organization (WHO) 2-level category with 70% diagnosis consistency [17]. Further differentiation of atypical and carcinoma was following a subset of WHO 6 categories. Figure 1 shows the non-atypical hyperplasia without expression of β-catenin and carcinoma with positive expression of β-catenin. The details are available in a Supplementary file.

### Sample size

The sample size was estimated with the effect size derived from the difference between the proportion of nuclear β-catenin expression between the premalignant endometrial hyperplasia and benign hyperplasia according to a systematic review [18]. With a 5% type 1 error, 95% power of the study, and a 20% dropout rate, at least 89 participants should be included in the study.

### Quantification of variables

Age and body mass index were presented as continuous data and its mean ± standard deviation. Further classification of body mass index following the Asian classification. Episodes of parity, miscarriage or abortus were presented with median and interquartile ranges. No specific quantification and discretization were made for other variables.

### Analysis

Descriptive statistics will elaborate on the characteristics of participants according to the pathology results. No missing data were imputed as all data included in this study should have complete responses. Normality tests along with the bivariate tests were conducted to assess the potential predictors. Differences between the two means were tested with independent t-test and Mann Whitney whereas three means were tested with Analysis of Variance/ ANOVA and Kruskal Wallis test. The Chi-square and Fischer Exact test concluded the association between categorical variables.

Before the regression test, the selection of parameters was based on the p-value of the bivariate test of at least less than 0.2. Backward selection was also performed case by case. As the level of pathology result was in three levels, hence, ordinal regression would be applied, assuming that the assumptions are met. Re-classification of the results was made by merging two levels to create the binary response. In terms of differentiating carcinoma, a subset of atypical and non-atypical was merged into one subset, whereas atypical and carcinoma were merged into one variable to assess the discriminant ability in the benign stage (non-atypical). Adjusted odds ratio (aOR) presented by the exponential B value (expB) along with the 95% confidence interval (CI) of expB was presented.

The Receiver Operating Characteristics (ROC) curve would assess the discriminant ability of β-catenin outcomes to carcinoma, and non-atypical classification without the presence of other predictors. The Area Under the Curve (AUC) and the cut-off point would be determined should the ROC curve not intersect and be located above the diagonal line.

To accommodate other factors in predicting the carcinoma and non-atypical classification, we applied an Artificial Neural Network and Decision Tree model to build the classifiers. Details of syntax commands were attached as a Supplementary file. We assessed the diagnostic performance of the model (sensitivity, specificity, AUC), as well as identifying the most important factors in the model. All analyses were conducted using Statistical Package for Social Science (SPSS) version 29.

### Possible bias

The reliability issue in assessing the pathology features was handled by involving two pathologists and one gynaecologist for clinical consideration. We also acknowledge the time-to-assessment was different between the samples however, a time constraint was set that all the samples should be interpreted according to a standard diagnostic time (within 24 h after immunohistochemistry staining).

## Results

**Fig. 2:**
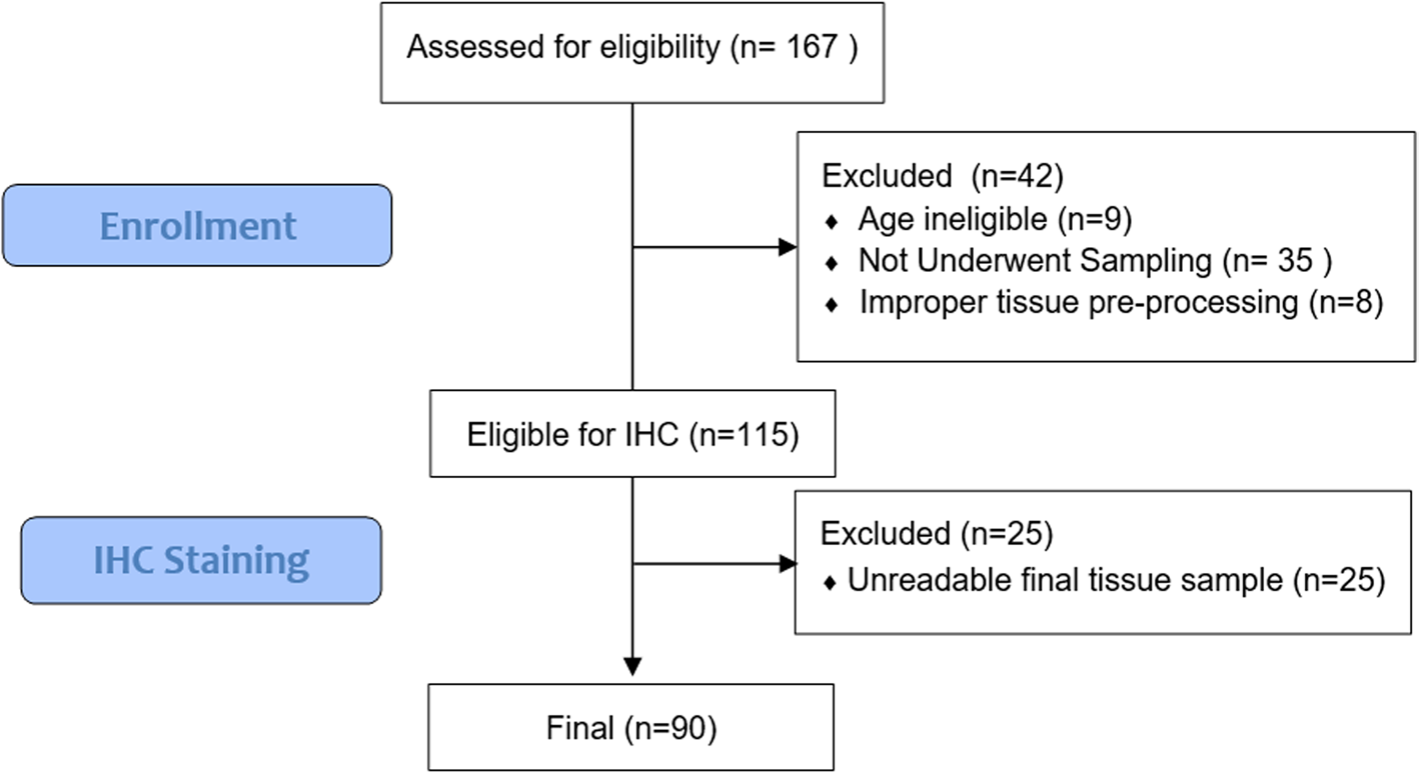
CONSORT participant’s flow

A total of 167 participants were screened for clinical assessment. Following the eligibility criteria, nine samples from patients who fell outside the target age group were excluded, leaving 158 individuals for pathology assessment. As 35 participants were unable to undertake sampling procedures (curettage or surgery), and eight samples could not proceed for hematoxylin-eosin staining, this selection left 115 participants for immunohistochemistry screening (fifteen participants repeated the sampling procedures). Under the criteria for immunohistochemistry (IHC) screening, 25 samples were not eligible for IHC reading, thus the final samples for assessment were 90 samples as presented in Fig. 2.

The mean age was 45.07 ± 9.07 (23–66 years old) with a median parity of 2 (Interquartile range 0–5) times. The majority of participants never experienced miscarriage or abortion (70%) and at normal body mass index (46.7%) although the mean BMI was 27.33 ± 5.22 kg/m^2^. The most frequent symptom reported by individuals was abnormal uterus bleeding (63.3%) with the most referred clinical diagnosis as Endometrial Hyperplasia (31.1%). Specimens were mostly obtained through surgical incision and/or hysterectomy (62.2%). In terms of pathology classification, 32 patients (35.6%) presented with non-atypical endometrial hyperplasia, 27 with atypical form (30.0%), and 31 (34.4%) demonstrated carcinoma presentation. Table 1 describes the characteristics of the participants according to the classification of pathological findings.

**Table 1:** Participant’s characteristics according to pathology results

Variables	Non-Atypical (*n*=32)	Atypical (*n*=27)	Endometrial Carcinoma (31)	***p***-value
Age*	44.97 ± 6.15	40.33 ± 10.33	49.29 ± 8.59	<0.001
Body Mass Index	26.12 ± 5.37	28.48 ± 4.79	27.57 ± 5.33	0.086
Parity Episode (Median)	2 (IQR 0-5)	2 (IQR 0-3)	3 (IQR 1-4)	0.101
Abortion Episode (Median)	0 (IQR 0-1)	1 (IQR-0-2)	1 (IQR 0-2)	0.308
Signs, Symptoms and Previous Intervention				
Vaginal Bleeding	12 (37.5%)	18 (66.7%)	15 (48.4%)	0.081
Abdominal Enlargement	5 (15.6%)	1 (3.7%)	13 (41.9%)	0.001
Mennorrhagia#	6 (18.8%)	6 (22.2%)	1 (3.2%)	0.065
Mennometroraghia	5 (15.6%)	8 (29.6%)	6 (19.3%)	0.404
Uterus Bleeding	20 (62.5%)	14 (51.8%)	23 (74.2%)	0.210
Abdominal Pain#	1 (3.1%)	2 (7.4%)	4 (12.9%)	0.330
Hormone Therapy#	2 (6.2%)	1 (3.7%)	2 (6.4%)	1.000
Previous Curretage#	0 (0%)	1 (3.7%)	1 (3.2%)	0.537
Referral Diagnosis				N/A
Abnormal Uterus Bleeding	16 (50%)	10 (37.0%)	1 (3.2%)	
Endometrial Hyperplasia	13 (40.1%)	11 (40.7%)	4 (12.9%)	
Ovarian Carcinoma	1 (3.1%)	1 (3.7%)	1 (3.2%)	
Adenomyosis	0 (0%)	1 (3.7%)	0 (0%)	
Endometrial Cysts	0 (0%)	2 (7.4%)	0 (0%)	
Myoma Uteri	6 (18.8%)	3 (11.1%)	2 (6.4%)	
Cervical Polyp	0 (0%)	1 (3.7%)	0 (0%)	
Endometrial Polyp	0 (0%)	1 (3.7%)	0 (0%)	
Endometrial Carcinoma	0 (0%)	1 (3.7%)	24 (77.4%)	
Cyst Torsion	0 (0%)	0 (0%)	1 (3.2%)	
β-Catenin				
Intensity	2.59 ± 0.49	2.96 ± 0.19	2.77 ± 0.42	0.004
Percentage	50.94 ± 22.63	68.15 ± 18.82	63.87 ± 23.48	0.008
Percentage Area Level	2.41 ± 0.49	2.78 ± 0.51	2.65 ± 0.61	0.008
H-Score	138.13 ± 75.79	204.07 ± 57.66	184.51 ± 68.30	0.003

Continuous data were tested with Kruskal Wallis except * (ANOVA); *IQR *Interquartile Range, Categorical data were tested with Chi-Square except for # (Fischer Exact). N/A means not applicable

Participants with endometrial carcinoma were significantly older compared to other types (*p* < 0.001). Interestingly, higher parity was observed in endometrial carcinoma although it was not significant (*p* = 0.101). Also, there were no significant differences in body mass index and abortion episodes (*p* > 0.05). Abdominal enlargement was seen more frequently in Endometrial Carcinoma (*p* = 0.001) but other symptom distributions were similar between the three classes. The majority of people referred with abnormal uterus bleeding were presented with non-atypical findings, and among 25 people referred with endometrial carcinoma, only one person had atypical findings. In terms of β-catenin expression, there was a significant difference between the three classes where β-catenin expression was higher in atypical cases, followed by carcinoma and non-atypical findings.

When considering the binary level of pathology results (Carcinoma versus Non-Carcinoma) and (Non-Atypical versus Typical and Carcinoma), different results could be seen. Aside from abdominal enlargement, menorrhagia (prolonged or heavy bleeding during the menstrual period) was significantly higher in non-carcinomatous patients (*p* = 0.030). The β-Catenin intensity, percentage, area and H-score were not significantly different between carcinoma and non-carcinoma (Supplementary Table 1), however, when considering non-atypical versus atypical plus carcinoma, along with BMI, β-Catenin outcomes (intensity, percentage, area and H-score) showed significant differences where the β-Catenin values were lower in non-atypical samples (Supplementary Table 2).

At first, ordinal logistic regression was planned to examine the interaction between independent variables and three levels of pathology results. However, the parallel lines test violated the ordinal regression assumption to which we applied Generalized Ordinal Logistic Regression. All variables with a p-value of less than 0.2 in Table 1 were included in the model presented in Table 2 except H-Score and Percentage area level to reduce collinearity with the percentage and intensity of β-catenin.

From Table 2, abdominal enlargement (aOR 6.703 95% CI 1.811–24.800 ) and percentage of β-catenin (aOR 1.024 95% CI 1.003–1.046) were the most significant predictors where it was associated with a severe level of pathology. In a different model (Supplementary Table 3), aside from abdominal enlargement (aOR 6.408 95% CI 1.738–23.632) H-score, which was calculated from both percentage and intensity of β-Catenin, had a significant effect on pathology level (aOR 1.009 95% 1.003–1.015). Supplementary Fig. 1 depicts the difference in the median of β-catenin and H-Score according to the pathology level.

**Table 2:** Generalized ordinal logistic regression of three pathology class

Parameter	B	Std. Error B	Sig.	Exp(B)	95% Confidence Interval for Exp(B)	
					Lower	Upper
Threshold						
Atypical	6.312	2.3428	0.007	551.279	5.587	54392.811
Carcinoma	7.824	2.4164	0.001	2499.001	21.925	284834.944
Vaginal Bleeding	0.607	0.4252	0.153	1.836	0.798	4.224
Abdominal Enlargement	1.902	0.6675	0.004	6.703	1.811	24.800
Menorrhagia	-0.066	0.5790	0.910	0.937	0.301	2.913
Age	0.038	0.0301	0.205	1.039	0.979	1.102
Parity	-0.023	0.2027	0.908	0.977	0.657	1.453
Body Mass Index	0.082	0.0463	0.075	1.086	0.992	1.189
Intensity β-Catenin	0.359	0.6293	0.568	1.432	0.417	4.917
Percentage β-Catenin	0.024	0.0106	0.024	1.024	1.003	1.046

Logistic regression with conditional backward was executed to assess the association of β-Catenin with binary classification (carcinoma versus non-carcinoma, and non-atypical versus atypical and carcinoma). The selection of parameters was based on variables with a p-value of less than 0.2 in Supplementary Tables 1 and 2.

Model three and four in Table 3 describes the significant association of β-Catenin percentage and H-score when comparing Non-Atypical versus Atypical and carcinoma where an increase of one per cent of β-Catenin expression was contributed to 1.032 times of developing later stage, adjusted by other factors (95% CI 1.010–1.054). A similar result was seen when considering the H-Score as the predictor where an increase of one unit of H-Score was associated with 1.011 times having a later stage of hyperplasia (95% CI 1.005–1.018). However, in models one and two when comparing carcinoma and non-carcinoma, increasing one year of age and the presence of clinical symptoms (abnormal uterus bleeding and abdominal enlargement) had a significant association with carcinoma but not with the BMI, β-catenin percentage and H-Score.

**Table 3:** Predictors of binary class

**Parameter Model 1** **Carcinoma Versus Non-Carcinoma**	**B**	**Standard Error B.**	***p*-value**	**Exp(B)**	**95% C.I.for EXP(B)**	
					**Lower**	**Upper**
BMI	0.101	0.058	0.081	1.107	0.988	1.240
Age	0.103	0.038	0.006	1.109	1.030	1.194
Uterus Bleeding	1.662	0.720	0.021	5.268	1.286	21.587
Abdominal Enlargement	2.851	0.800	<0.001	17.299	3.605	83.018
% β-Catenin	0.019	0.013	0.142	1.019	0.994	1.045
Adjusted R^2^= 0.414						
**Parameter Model 2** **Carcinoma Versus Non-Carcinoma**	**B**	**Standard Error B.**	***p*-value**	**Exp(B)**	**95% C.I.for EXP(B)**	
					**Lower**	**Upper**
BMI	0.091	0.057	0.111	1.095	0.979	1.224
Age	0.099	0.037	0.007	1.104	1.027	1.187
Uterus Bleeding	1.669	0.723	0.021	5.308	1.288	21.886
Abdominal Enlargement	2.801	0.801	<0.001	16.455	3.426	79.035
H- Score β-Catenin	0.005	0.004	0.228	1.005	0.997	1.013
Adjusted R^2^= 0.406						
**Parameter Model 3** **Non-Atypical Versus Atypical + Carcinoma**	**B**	**Standard Error B.**	***p*-value**	**Exp(B)**	**95% C.I.for EXP(B)**	
					**Lower**	**Upper**
BMI	0.077	0.048	0.106	1.080	0.984	1.185
Vaginal Bleeding	0.925	0.490	0.059	2.523	0.966	6.589
% β-Catenin	0.031	0.011	0.004	1.032	1.010	1.054
Constant	-3.757	1.516	0.013	0.023		
Adjusted R^2^= 0.214						
**Parameter Model 4** **Non-Atypical Versus Atypical + Carcinoma**	**B**	**Standard Error B.**	***p*-value**	**Exp(B)**	**95% C.I.for EXP(B)**	
					**Lower**	**Upper**
Vaginal Bleeding	0.834	0.487	0.087	2.303	0.887	5.982
H Score β-Catenin	0.011	0.003	0.001	1.011	1.005	1.018
Constant	-1.680	0.659	0.011	0.186		
Adjusted R^2^= 0.215						

Considering these logistic models as a classifier, all models possess a good specificity of over 80% but not with sensitivity (less than 55%) to predict two different binary stagings. Further ROC (Supplementary Fig. 2) analysis of both β-catenin percentage for carcinoma, and H-Score for Non-Atypical show insignificant discriminant ability as the ROC curve intersects with a diagonal line, hence misspecification of the cases occurred. This indicates that using β-catenin value only to screen carcinoma and non-carcinoma, as well as non-atypical and other types is not valid. Suppose that the AUC values were significant, the ideal cut-off of β-catenin and H-score with higher sensitivity and specificity (at least 50%) to distinguish non-atypical from other stages was 55% (sensitivity 75.9% specificity 59.4%) and 130 (sensitivity 82.8% specificity 56.2%), whereas there was no ideal cut-off of β-catenin percentage and H-score for differentiating carcinoma.

Two models based on Artificial Neural Networks and Decision Trees were made for binary classification for carcinoma and non-atypical class. The sensitivity and specificity of the model to distinguish carcinoma based on model 1 in Table 3 were improved from the logistic regression model in testing data (sensitivity 70.0% and 88.2%). All predictors had a normalized importance score above 50% with age as the most important factor (Supplementary Table 4). The neural network using model 2 achieved a lower sensitivity but higher specificity (sensitivity 60.0% and 94.1%), however, the H-Score only had a normalized importance score of 22.8% (Supplementary Table 5). The neural network to distinguish between non-atypical and atypical + carcinoma derived from model 3 exhibits sensitivity of 80.0% and 60% in testing data with % β-catenin as the most important factor (Supplementary Table 6). However, the neural network from model 4 has a lower specificity (45.5%) but better sensitivity (87.5%) where H-score was the most essential factor (Supplementary Table 7).

A decision tree of model 3 to distinguish between non-atypical and atypical + carcinoma showed a higher sensitivity of 94.8% but a very low specificity (40.8%) with a cut-off of % β-catenin score of 55 (Supplementary Table 8). Moreover, the decision tree model from model 4 yielded a sensitivity of 89.7% and specificity of 50% when using the cut-off H-Score of 110 (Supplementary Table 9). Since β-catenin percentage and H-score were not associated with carcinoma, therefore, no decision tree model was made. The summary of performance is concluded in Supplementary Table 10.

## Discussion

### Summary

Our study identified the association between β-catenin and the level of endometrial hyperplasia, adjusted by other factors. Moreover, combined with clinical symptoms and individual factors using advanced classifiers, β-catenin can distinguish benign lesions (non-atypical) and later stages, as well as carcinoma, thus, increasing the precision and confidence in pathology assessment of endometrial hyperplasia.

### Association of clinical symptoms and Individual factors with β-catenin and pathology grading

Age was significant when differentiating carcinoma from non-carcinoma, This result is linear with a study from Israel, which stated that older women demonstrated prevalent high-risk histologies [19]. However, the Pearson correlation shows a non-significant correlation between age and β-catenin intensity, percentage and H-score (*p* > 0.05) (supplementary Table 11), meaning that multiple age-related factors are influencing the carcinoma progression and not solely due to β-catenin expression in endometrium.

Body mass index was an insignificant predictor of pathology class and its addition to the predictive model did not contribute to a significant association as shown in Table 3. The BMI trajectories (rather than current BMI) particularly in adulthood were more important in endometrial cancer risk where longer exposure to overweight and obesity contributed to an increased risk of endometrial cancer [20]. Further analysis in this study revealed that β-catenin intensity was positively correlated with BMI (Rs 0.251 *p* = 0.017 in supplementary Table 10), thus, when a person is exposed to obesity for a longer time, the β-catenin exposure, as well as abnormal Wnt/β-catenin signalling, also occur [21].

The initial episode of menorrhagia is assumed to be an impact of estrogen and progesterone imbalance activity. Through its receptor ERα (ESR1) and ERβ (ESR2), estrogen induces the proliferation of stromal cells and endometrial epithelial cells, whereas progesterone performs a counteracting effect of estrogen [22]. There is a connection between β-catenin and estrogen which may contribute to the development of the endometrium. Estrogen was found to be affecting β-catenin by upregulating β-catenin mRNA and protein expression mediated by Estrogen Receptors in stromal cells, activating the Wnt/β-catenin signalling pathway and, stimulating ESR1 and β-catenin co-localization in the nucleus in of stromal cells in normal circumstances [23].

A study shows that estrogen and progesterone were exclusively expressed in stromal of non-atypical hyperplasia, compared to β-catenin which was expressed in non-atypical and atypical hyperplasia [24]. Menorrhagia was insignificantly associated with the pathology level in our study but was frequently observed in non-atypical and atypical findings. However, a study in the United States shows an insignificant association of menorrhagia to uterine cancer among patients who visited the clinic with gynaecology problems (aOR 1.2 with 95% CI 0.7–2.2) [25]. Interestingly, further analysis in our study shows no significant difference in β-catenin values and menorrhagia (*p* > 0.05) (supplementary Table 11).

Frequent episodes of parity were associated with lower endometrial cancer risk as stated in a meta-analysis [26] and our study revealed an insignificant dose-response protection (0.977 95% CI 0.657–1.453 in Table 2). However, there was no significant correlation between the number of parity and β-catenin values (*p* > 0.05 supplementary Table 10). It is important to identify the difference in the expression of genes related to endometrial proliferation between nulliparous and multiparous women, particularly the presence of abnormal Wnt/β-catenin signalling.

### The β-catenin values show a better prognostic factor for the benign stage, rather than carcinoma

A good screening model should possess a higher sensitivity and β-catenin values along with clinical symptoms and show a better discriminant ability to distinguish non-atypical lesions as these models show at least 80% sensitivity. However, higher specificity (at least 50%) was achieved only with neural network model 3 and decision tree model 4. A study involving neural networks to predict a class of tuberculosis resistance demonstrates that neural networks outperform another classifier model when combining clinical symptoms, demography features and laboratory results, whereas decision trees possess higher sensitivity but very low specificity [27]. In this study. β-catenin percentage of 55 has a nonsignificant diagnosis performance in the ROC curve (sensitivity 75.9% specificity 59.4%), and the sensitivity improved with neural network model Sensitivity (80.0% and specificity 60%). The decision tree model with a cut-off of 55% β-catenin has a higher sensitivity but very low specificity (94.8%). In the neural network, the performance was similar to ROC (Sensitivity of 80.0% and specificity of 60%) meaning that neural networks outperform other models. A pathologist could apply this cut-off of 55 to identify any focal lesion that shows atypical or focal carcinoma. Another study demonstrated the association between β-catenin expression as a predictor for poor prognosis [28]. In short, β-catenin expression, along with suggestive symptoms shows a promising discriminant ability to distinguish non-atypical with advanced levels of pathology.

### Strength and limitations

This study applied a standardized preparation of samples from curettage and surgical procedure and the samples were processed according to a standard β-catenin immunohistochemistry staining. Moreover, robust statistical methods were implemented to derive the conclusions.

Despite being justified by statistical estimation, the sample size should be increased to maximize the model building and model performance. Furthermore, prospective testing with new patients should be done to identify the consistency of diagnostic performance. In addition, the generalizability of the study is limited as this study was done in a single centre that supervised the two hospitals, although this centre accepts referred cases from the eastern part of Indonesia.

There is a question of whether hormonal therapy may affect the expression of β-catenin in endometrial tissue. Estradiol possesses a potential role in Wnt/β-catenin signalling by affecting the transcription of Wnt/β-catenin target genes [29] and enhancing the β-catenin intracellular stabilization and translocation to the nucleus through indirect crosstalking of PI3K2 pathway and canonical Wnt signalling, whereas progestogen inhibit the Wnt/β-catenin signalling by enhancing DKK-1 in endometrium [30]. Additional analysis shows a significant difference in the percentage of β-catenin between those who received hormonal therapy and not (38% versus 61% *p* < 0.05). However, the distribution of the participants according to hormonal therapy was similar across pathology levels, thus this factor may not be a potential confounder.

Diabetes and hypertension were associated with endometrial carcinoma. However, this study did not objectively assess diabetes and hypertension status, thus the expression of β-catenin among participants with the chronic disease could not be identified and the potential confounding issue was not explored.

Lastly, there are some other potential predictors including Cyclooxigenase-2 (COX2) expression for endometrial carcinoma [31], but this is beyond the scope of the study and further research to prove the incremental diagnostic value of COX2 and β-catenin should be done.

## Conclusion

The β-catenin expression, particularly percentage and H-Score is a good predictor to differentiate carcinoma, or even earlier stage when combined with clinical symptoms and demographic parameters. Furthermore, BMI was associated with β-catenin intensity and therefore, BMI reduction may play a role in reducing the risk of disease progression. This study also proposes a new cut-off for β-catenin percentage (55) that could discriminate non-atypical conditions from later stages although a prospective test is needed.

### Supplementary Information

**Additional file 1. **Supplementary file.

**Additional file 2: Supplementary Table 1.** Characteristics of participants based on carcinoma and non-carcinoma findings. **Supplementary Table 2.** Characteristics of participants based on non-atypical and later stages findings. **Supplementary Table 3.** Generalized ordinal logistic regression of three pathology classes considering the h-score of β-catenin. **Supplementary Table 4.** Model structure of artificial neural network in predicting carcinoma using % of beta-catenin. **Supplementary Table 5.** Model structure of artificial neural network in predicting carcinoma using h-score of beta-catenin. **Supplementary Table 6.** Model structure of artificial neural network in predicting non-atypical finding using % of beta-catenin. **Supplementary Table 7.** Model structure of artificial neural network in predicting non-atypical finding using h-score of beta-catenin. **Supplementary Table 8.** Model structure of decision tree in predicting non-atypical finding using % beta-catenin. **Supplementary Table 9.** Model structure of decision tree in predicting non-atypical finding using h-score of beta-catenin. **Supplementary Table 10.** Summary of model performance. **Supplementary Table 11.** Additional analysis.

**Additional file 3: Supplementary Figure 1. **Violin plots of β-catenin percentage and H-Score according to the three-level of histopathology. The median of %β-catenin and H-Score were higher in Atypical, followed by carcinoma and non-atypical hyperplasia. **Supplementary Figure 2.** ROC Curve of β-Catenin and H-Score in Differentiating Carcinoma, and Non-Atypical. Not that all ROC curve intersect the diagonal line, thus conclusion should not be made when considering beta-catenin as a single discriminant factor.

## Data Availability

The datasets used and/or analysed during the current study are available from the corresponding author upon reasonable request.

## Abbreviations

ANOVA: Analysis of Variance
aOR: Adjusted Odds Ratio
AUC: Area Under Curve
BMI: Body Mass Index
CD10: Cluster of Differentiation 10
CI: Confidence Interval
CTNNB1: Catenin Beta-1
COX-2: Cyclooxygenase-2
DAB: Diaminobenzidine
DKK-1: Dickkopf-1
Erα: Estrogen Receptor Alpha
Erβ: Estrogen Receptor Beta
Exp(B): Exponential of B
HRP: Horseradish Peroxidase
ICD: International Classification of Disease
IHC: Immunohistochemistry
IQR: Interquartile Range
mRNA: Messenger Ribonucleic Acid
PBS: Phosphate Buffer Saline
PI3K2: Phosphoinositide-3-Kinase 2
ROC: Receiver Operating Characteristics
TBS: Tris Buffer Saline
WHO: World Health Organization

## Declarations

As the data was part of a routine medical procedure, the hospital also gave consent in a statement as follows, “As our hospital is one of the centres for medical education, in certain parts of your care, there will be an involvement of students who are equipped with sufficient clinical knowledge to observe and perform medical procedures and further examination when deemed necessary. Moreover, our hospital also serves as a clinical research unit that will employ and utilise the data stored in the medical record for medical training and research. This is in line with the Health Ministerial Regulation number 269 year 2008 regarding medical records…”. The aforementioned IRB also granted the waiver for additional informed consent following the regulation by the Ministry of Health.

## References

[1] Kurman RJ, Norris HJ (1982). Evaluation of criteria for distinguishing atypical endometrial hyperplasia from well-differentiated carcinoma. Cancer.

[2] Trimble CL, Kauderer J, Zaino R, Silverberg S, Lim PC, Burke JJ (2006). Concurrent endometrial carcinoma in women with a biopsy diagnosis of atypical endometrial hyperplasia: a Gynecologic Oncology Group study. Cancer.

[3] Braun MM, Overbeek-Wager EA, Grumbo RJ (2016). Diagnosis and management of Endometrial Cancer. Am Fam Physician.

[4] Jeong JY, Hwang SO, Lee B, Kim K, Kim YB, Park SH (2020). Risk factors of progression to endometrial cancer in women with endometrial hyperplasia: a retrospective cohort study. PLoS ONE.

[5] Rodriguez AM, Polychronopoulou E, Hsu E, Shah R, Lamiman K, Kuo YF (2021). Factors associated with endometrial cancer and hyperplasia among middle-aged and older hispanics. Gynecol Oncol.

[6] Zhao J, Hu Y, Zhao Y, Chen D, Fang T, Ding M (2021). Risk factors of endometrial cancer in patients with endometrial hyperplasia: implication for clinical treatments. BMC Womens Health.

[7] Raglan O, Kalliala I, Markozannes G, Cividini S, Gunter MJ, Nautiyal J (2019). Risk factors for endometrial cancer: an umbrella review of the literature. Int J Cancer.

[8] Boeckstaens S, Dewalheyns S, Heremans R, Vikram R, Timmerman D, Van den Bosch T (2020). Signs and symptoms associated with Uterine cancer in pre- and postmenopausal women. Heliyon.

[9] Tian X, Liu Z, Niu B, Zhang J, Tan TK, Lee SR (2011). E-cadherin/β-catenin complex and the epithelial barrier. J Biomed Biotechnol.

[10] Parrish ML, Broaddus RR, Gladden AB (2022). Mechanisms of mutant β-catenin in endometrial cancer progression. Front Oncol.

[11] McConechy MK, Ding J, Senz J, Yang W, Melnyk N, Tone AA (2014). Ovarian and endometrial endometrioid carcinomas have distinct < em > CTNNB1 and < em > PTEN mutation profiles. Mod Pathol.

[12] Schlosshauer PW, Ellenson LH, Soslow RA (2002). Beta-catenin and E-cadherin expression patterns in high-grade endometrial carcinoma are associated with histological subtype. Mod Pathol.

[13] Jeong JW, Lee HS, Franco HL, Broaddus RR, Taketo MM, Tsai SY (2009). beta-catenin mediates glandular formation and dysregulation of beta-catenin induces hyperplasia formation in the murine uterus. Oncogene.

[14] Travaglino A, Raffone A, Saccone G, De Luca C, Mollo A, Mascolo M (2019). Immunohistochemical nuclear expression of β-Catenin as a Surrogate of CTNNB1 exon 3 mutation in Endometrial Cancer. Am J Clin Pathol.

[15] Barker N, Born M, Vincan E (2008). Detection of β-Catenin localization by immunohistochemistry. Wnt signaling: pathway methods and mammalian models.

[16] Jung C-K, Jung J-H, Lee A, Lee Y-S, Choi Y-J, Yoon S-K (2008). Diagnostic use of nuclear β-catenin expression for the assessment of endometrial stromal tumors. Mod Pathol.

[17] Sobczuk K, Sobczuk A (2017). New classification system of endometrial hyperplasia WHO 2014 and its clinical implications. Przeglad Menopauzalny = Menopause Review.

[18] Travaglino A, Raffone A, Saccone G, Mascolo M, D’Alessandro P, Arduino B (2019). Nuclear expression of β-catenin in endometrial hyperplasia as marker of premalignancy. APMIS.

[19] Hag-Yahia N, Gemer O, Eitan R, Raban O, Vaknin Z, Levy T (2021). Age is an Independent predictor of outcome in endometrial cancer patients: an Israeli Gynecology Oncology Group cohort study. Acta Obstet Gynecol Scand.

[20] Dalmartello M, Vermunt J, Negri E, Levi F, La Vecchia C (2022). Adult lifetime body mass index trajectories and endometrial cancer risk. BJOG.

[21] Chen N, Wang J (2018). Wnt/β-Catenin signaling and obesity. Front Physiol.

[22] Hapangama DK, Kamal AM, Bulmer JN (2015). Estrogen receptor β: the guardian of the endometrium. Hum Reprod Update.

[23] Xiong W, Zhang L, Yu L, Xie W, Man Y, Xiong Y (2015). Estradiol promotes cells invasion by activating β-catenin signaling pathway in endometriosis. Reproduction.

[24] Chatzipantelis P, Koukourakis M, Balaska K, Giatromanolaki A (2022). Endometrial stromal expression of ER, PR, and B-Catenin toward differentiating Hyperplasia Diagnoses. Int J Surg Pathol.

[25] Endometrial Cancer Associated Symptoms (2016). A case-control study. J Women’s Health.

[26] Wu Q-J, Li Y-Y, Tu C, Zhu J, Qian K-Q, Feng T-B (2015). Parity and endometrial cancer risk: a meta-analysis of epidemiological studies. Sci Rep.

[27] Herman B, Sirichokchatchawan W, Pongpanich S, Nantasenamat C (2021). Development and performance of CUHAS-ROBUST application for pulmonary rifampicin-resistance Tuberculosis screening in Indonesia. PLoS ONE.

[28] Deng L, Liang H, Han Y (2020). Cyclooxygenase-2 and β-Catenin as potential diagnostic and prognostic markers in Endometrial Cancer. Front Oncol.

[29] Wang Y, van der Zee M, Fodde R, Blok LJ (2010). Wnt/Β-catenin and sex hormone signaling in endometrial homeostasis and cancer. Oncotarget.

[30] Tulac S, Overgaard MT, Hamilton AE, Jumbe NL, Suchanek E, Giudice LC (2006). Dickkopf-1, an inhibitor of wnt signaling, is regulated by progesterone in human endometrial stromal cells. J Clin Endocrinol Metab.

[31] Steinbakk A, Gudlaugsson E, Aasprong OG, Skaland I, Malpica A, Feng W (2011). Molecular biomarkers in endometrial hyperplasias predict cancer progression. Am J Obstet Gynecol.

