# Supplementary material for: Risk-stratification machine learning model using demographic factors, gynaecological symptoms and β-catenin for endometrial hyperplasia and carcinoma: a cross-sectional study
Source: BMC Womens Health. 2023 Nov 27;23:627. doi: 10.1186/s12905-023-02790-6 (PMC10680196; doi:10.1186/s12905-023-02790-6)
Supplement: Supplementary file 1 — Additional file 1. Supplementary file. [file 12905_2023_2790_MOESM1_ESM.docx]

**Supplementary File**

1. Standard interpretation

a. Percentage of stained area (chromic brownish area)

- No stained glandural or tumor cells: 0
- Stained less than 10% : 1+
- Stained between 10-50% : 2+
- Stained more than 50% : 3+

b. Intensity

- No stained glandural or tumor cells: 0
- Weakly stained or only visible at 40x magnification : 1+
- Moderately stained or visible at 20x magnification : 2+
- Clearly stained or visible at 10x magnification : 3+

Image Samples

- Endometrial hyperplasia without atypia
  - Architecture:
    - Closely packed glands such that gland to stroma ratio is > 3:1 but stroma is still present between glandular basement membranes (however minimal)
    - Variation in gland size with cystic dilatation or irregular luminal contours (budding, angulation, invagination, outpouching, papillary projections)
    - Associated with stromal breakdown
    - Increased volume of endometrial tissue on biopsy / curetting is typical but NOT required for diagnosis
  - Cytologic features:
    - Reminiscent of normal proliferative endometrium with pseudostratified, mitotically active, elongated columnar cells
    - Can show mild cellular enlargement but retain smooth nuclear contours without distinct nucleoli
    - Metaplastic changes common (eosinophilic, papillary syncytial, squamous morular, mucinous, ciliated)
- Atypical and Endometrial Carcinoma
  - Architecture:
    - Similar to the spectrum described above for hyperplasia without atypia
  - Cytologic features:
    - Enlarged, rounded and irregular nuclear contours
    - Prominent, enlarged nucleoli with coarse and vesicular chromatin
    - Occasionally, cytoplasmic eosinophilia imparts a distinct low power appearance
    - Stratified cells demonstrating loss of polarity with respect to basement membrane
    - Metaplastic changes can be seen

**Non-Atypic**

1. Intensity and Percentage Level 0


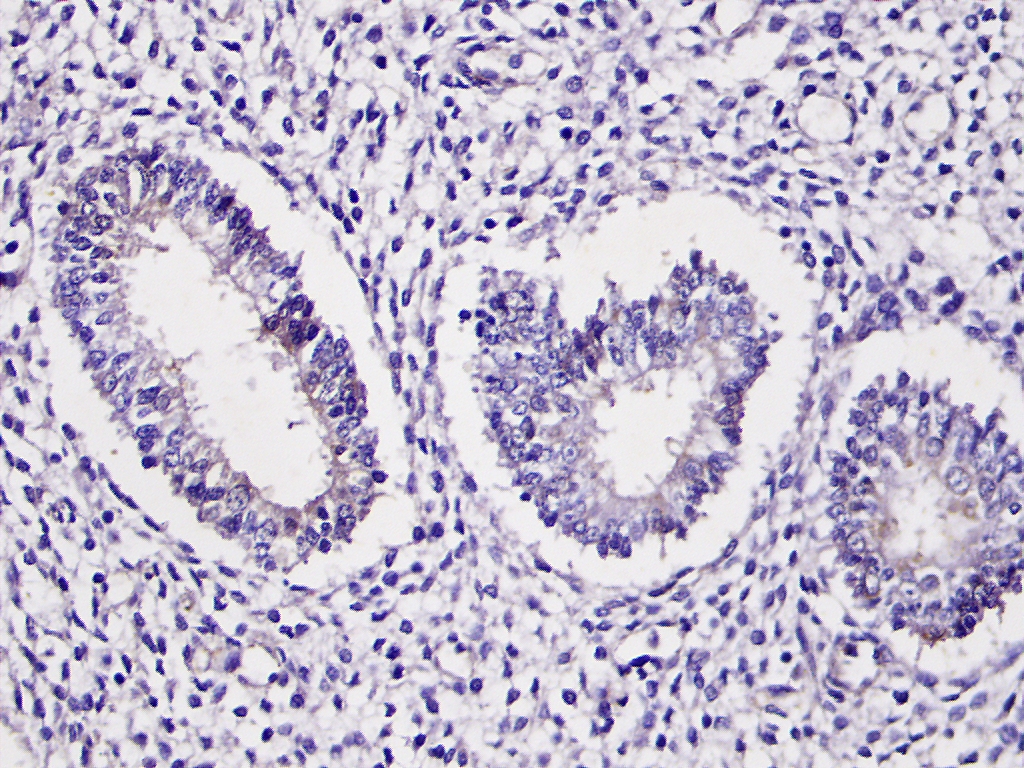


2. Intensity and Percentage Level 1


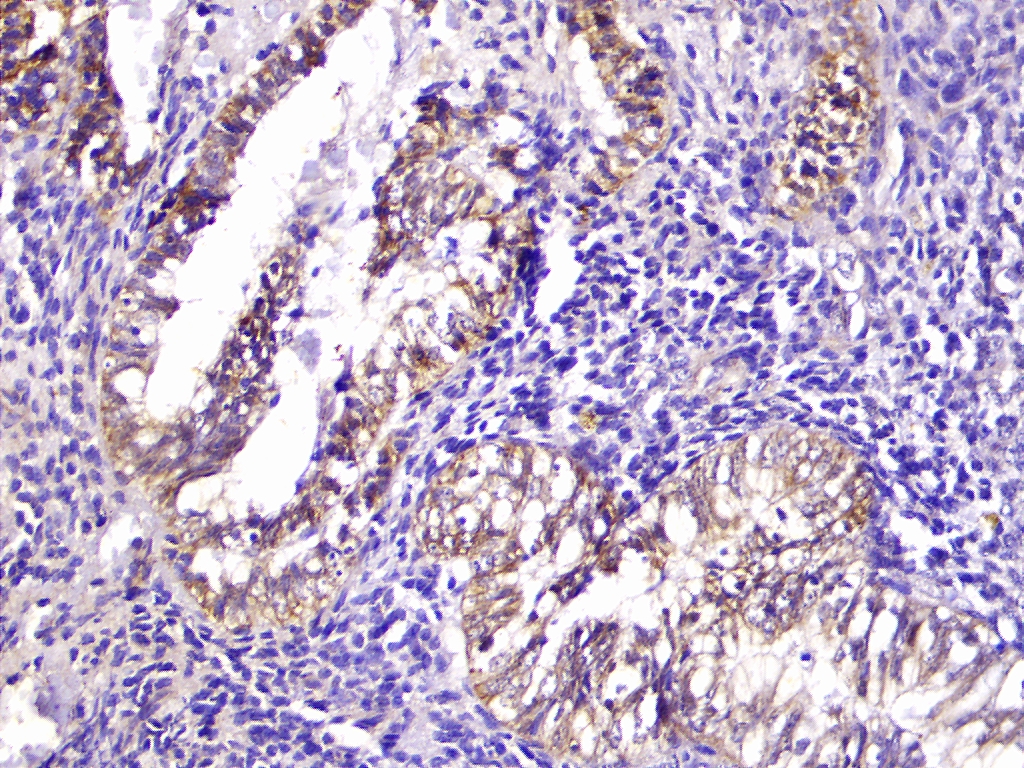


3. Intensity and Percentage Level 2


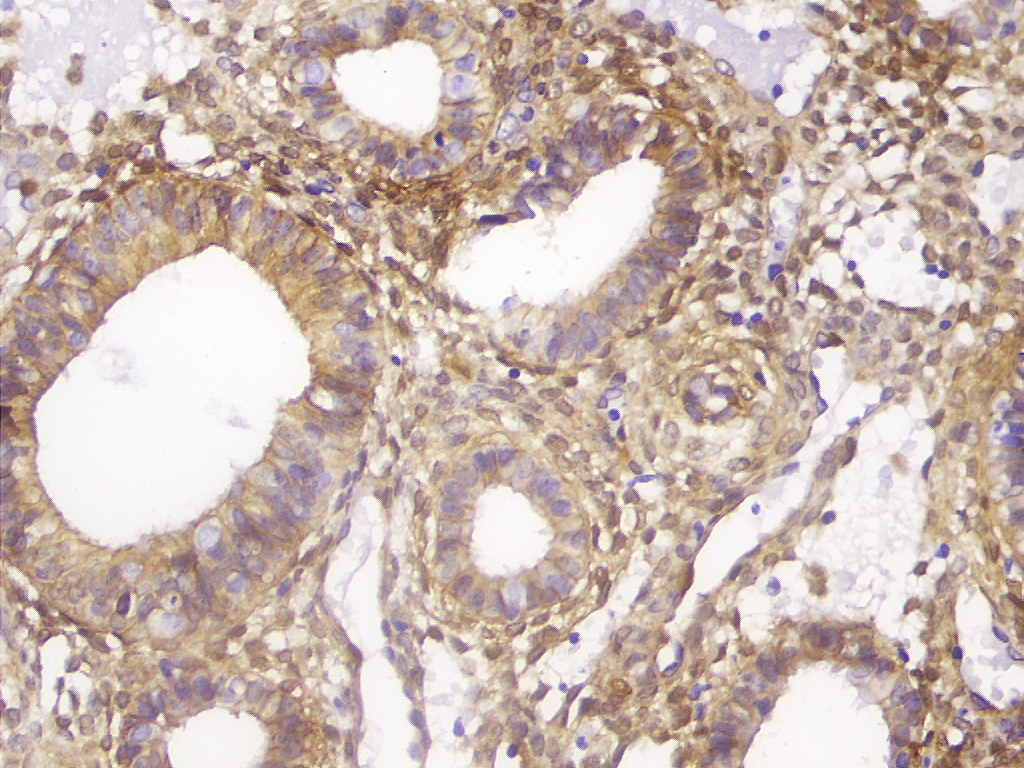


4. Intensity and Percentage Level 3


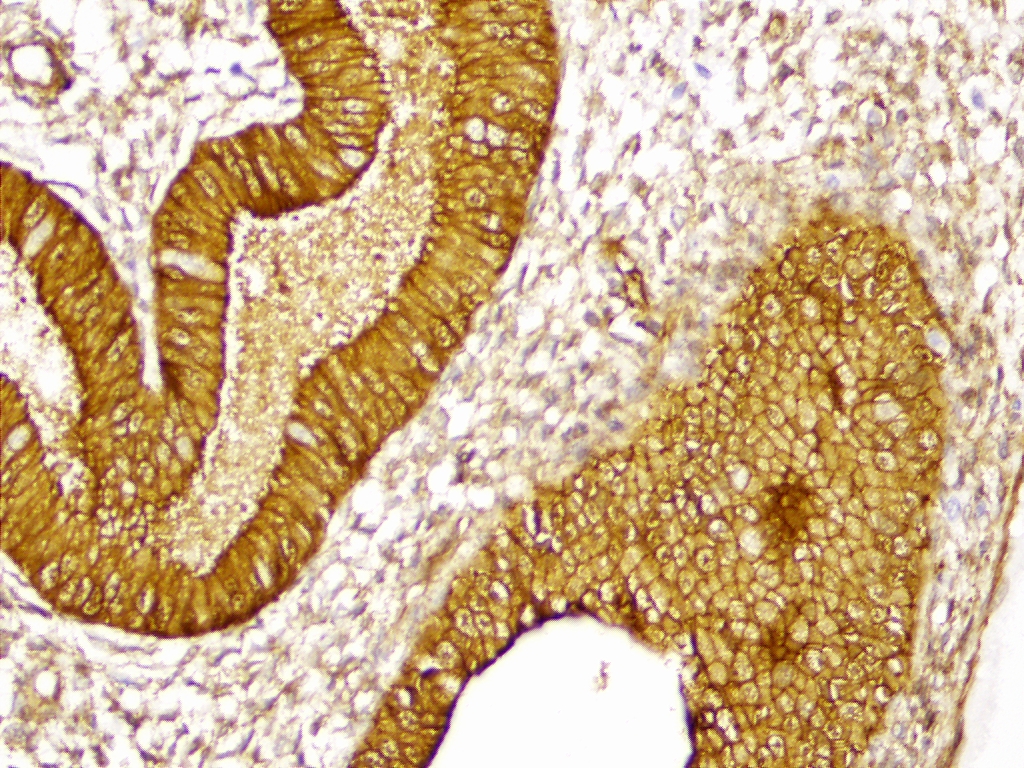


**Atypical**

1. Intensity and Percentage Level 0


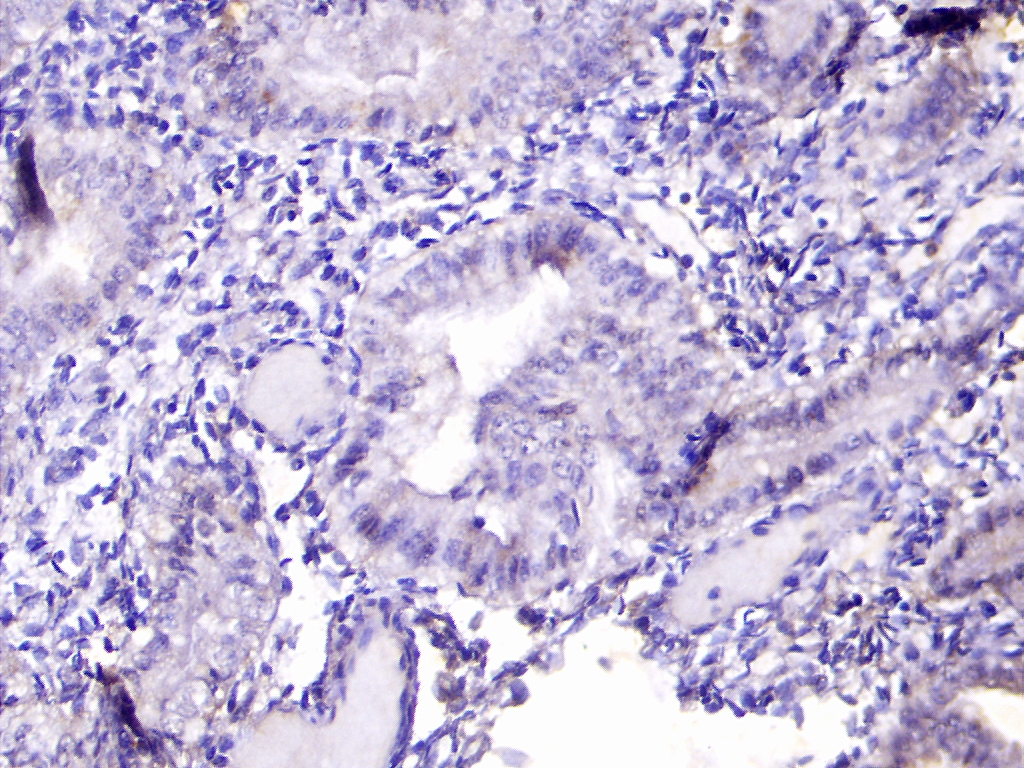


2. Intensity and Percentage Level 1


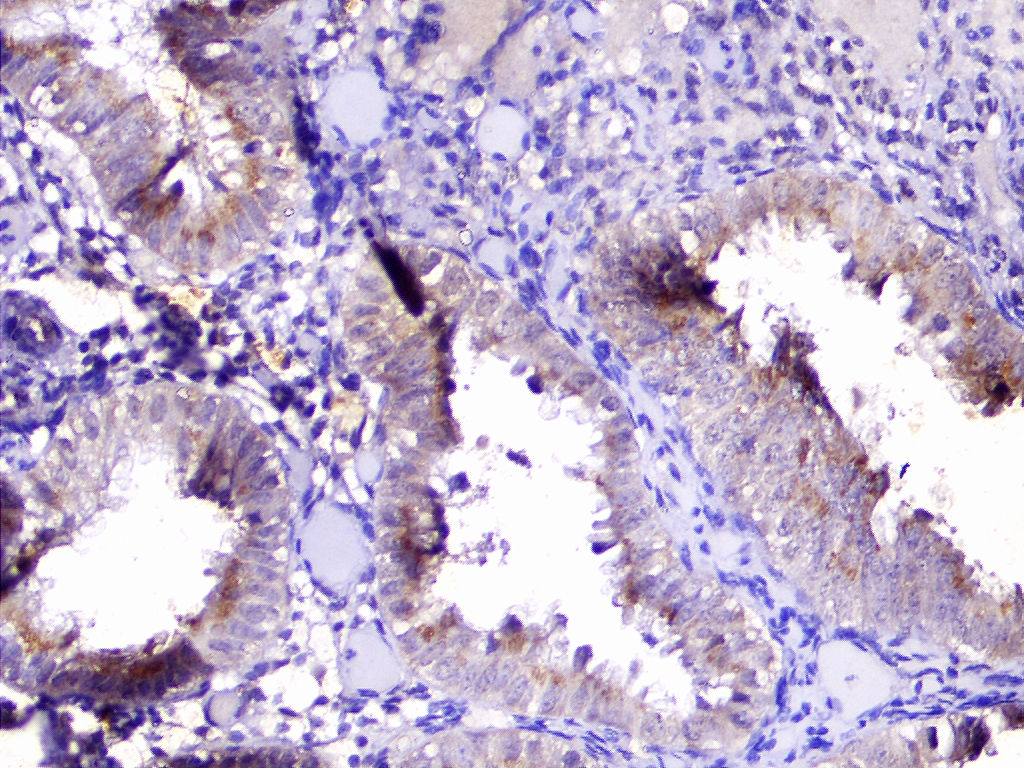


3. Intensity and Percentage Level 2


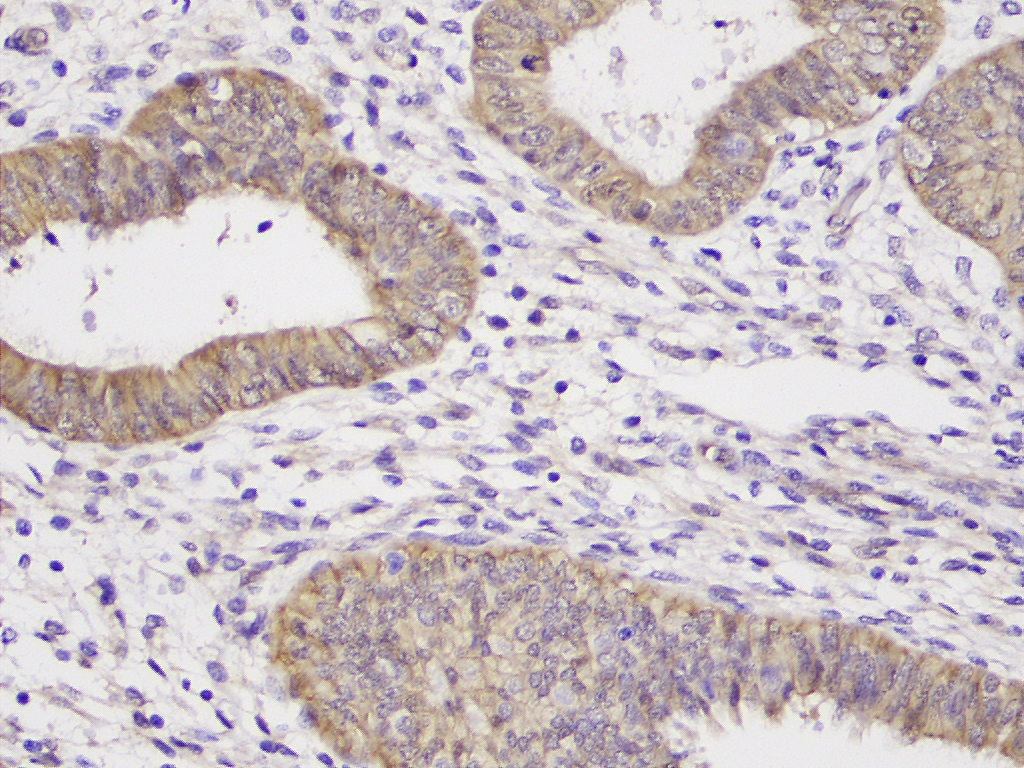


4. Intensity and Percentage Level 3


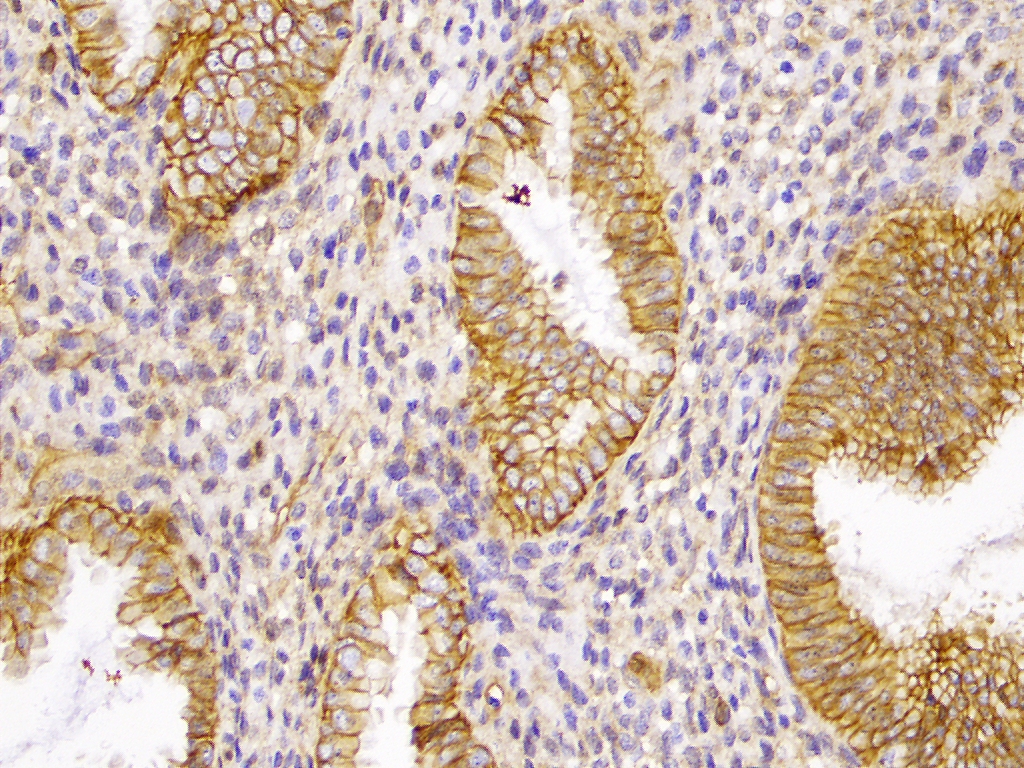


**Carcinoma**

1.Intensity and Percentage Level 0


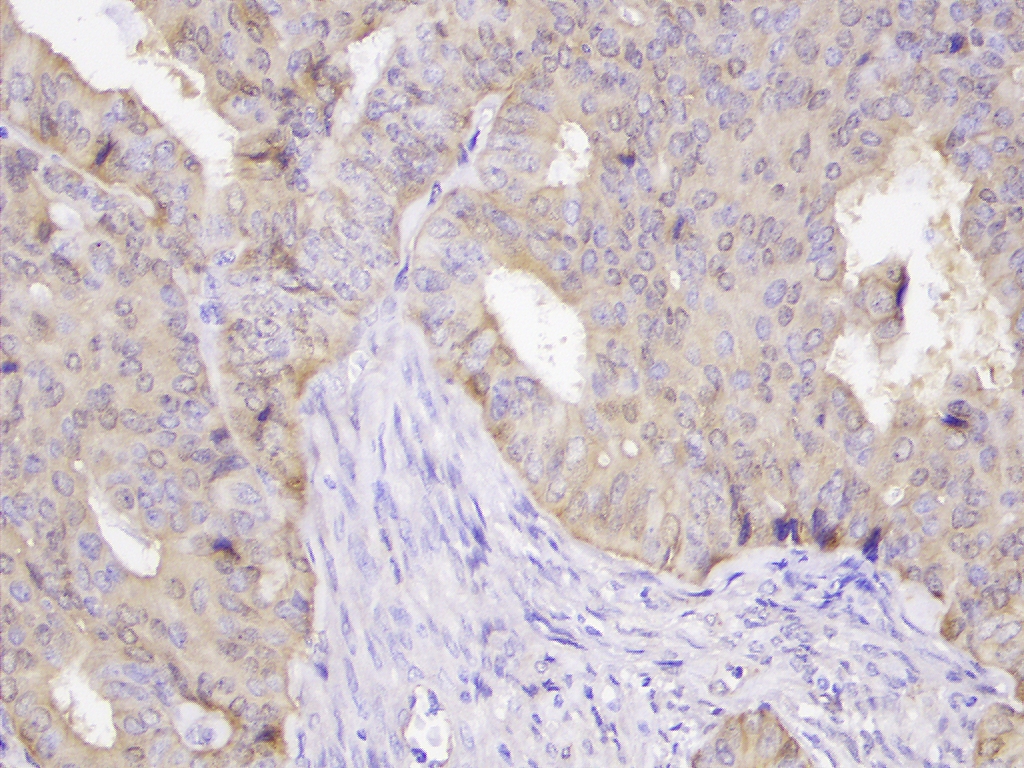


2.Intensity and Percentage Level 1


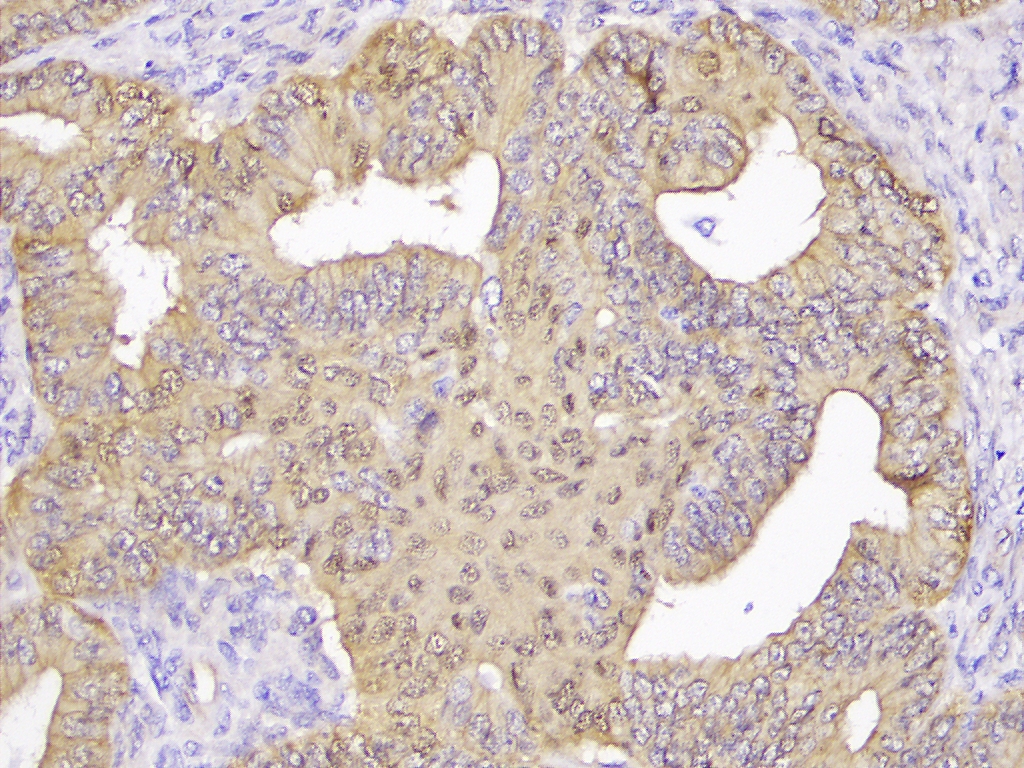


3.Intensity and Percentage Level 2


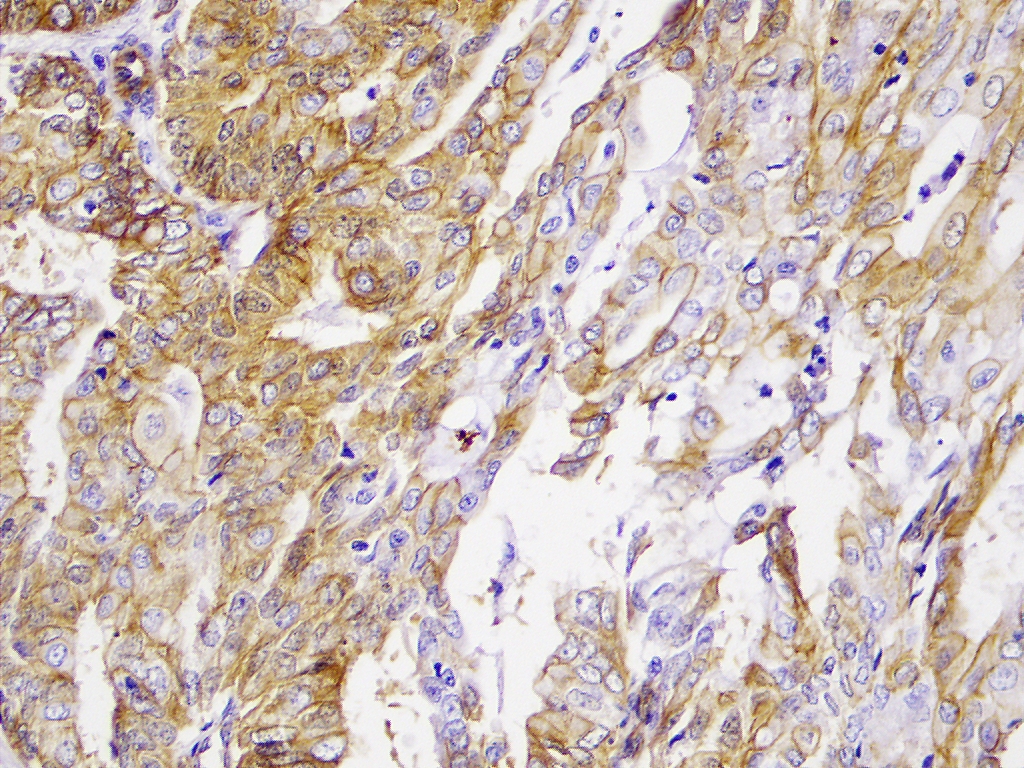


4.Intensity and Percentage Level 3


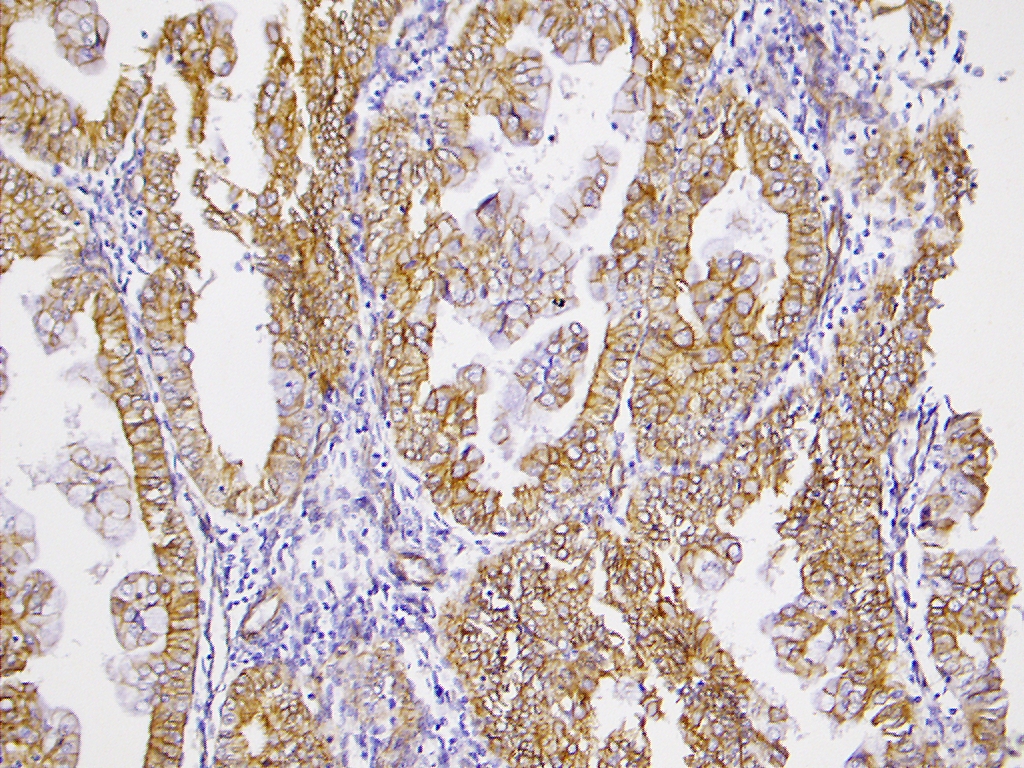


**SYNTAX MODEL**

***CARCINOMA VERSUS NON CARCINOMA***

**Model 1**

**SET SEED 123.**

***Multilayer Perceptron Network.**

**MLP binaryclass (MLEVEL=N) BY Abn_ute_bleed Abd_enl WITH Age percentBCat BMI**

**/RESCALE COVARIATE=STANDARDIZED**

**/PARTITION VARIABLE=Data**

**/ARCHITECTURE AUTOMATIC=YES (MINUNITS=1 MAXUNITS=50)**

**/CRITERIA TRAINING=BATCH OPTIMIZATION=SCALEDCONJUGATE LAMBDAINITIAL=0.0000005**

**SIGMAINITIAL=0.00005 INTERVALCENTER=0 INTERVALOFFSET=0.5 MEMSIZE=1000**

**/PRINT CPS NETWORKINFO SUMMARY CLASSIFICATION SOLUTION IMPORTANCE**

**/PLOT NETWORK ROC GAIN LIFT PREDICTED**

**/STOPPINGRULES ERRORSTEPS= 1 (DATA=AUTO) TRAININGTIMER=ON (MAXTIME=15) MAXEPOCHS=AUTO**

**ERRORCHANGE=1.0E-4 ERRORRATIO=0.001**

**/MISSING USERMISSING=EXCLUDE .**

**Model 2**

**SET SEED 123.**

***Multilayer Perceptron Network.**

**MLP binaryclass (MLEVEL=N) BY Abn_ute_bleed Abd_enl WITH Age BMI HscoreBC**

**/RESCALE COVARIATE=STANDARDIZED**

**/PARTITION VARIABLE=Data**

**/ARCHITECTURE AUTOMATIC=YES (MINUNITS=1 MAXUNITS=50)**

**/CRITERIA TRAINING=BATCH OPTIMIZATION=SCALEDCONJUGATE LAMBDAINITIAL=0.0000005**

**SIGMAINITIAL=0.00005 INTERVALCENTER=0 INTERVALOFFSET=0.5 MEMSIZE=1000**

**/PRINT CPS NETWORKINFO SUMMARY CLASSIFICATION SOLUTION IMPORTANCE**

**/PLOT NETWORK ROC GAIN LIFT PREDICTED**

**/STOPPINGRULES ERRORSTEPS= 1 (DATA=AUTO) TRAININGTIMER=ON (MAXTIME=15) MAXEPOCHS=AUTO**

**ERRORCHANGE=1.0E-4 ERRORRATIO=0.001**

**/MISSING USERMISSING=EXCLUDE .**

**SET SEED 123.**

***Multilayer Perceptron Network.**

**MLP binaryclass (MLEVEL=N) BY Abn_ute_bleed Abd_enl WITH Age percentBCat**

**/RESCALE COVARIATE=STANDARDIZED**

**/PARTITION VARIABLE=Data**

**/ARCHITECTURE AUTOMATIC=YES (MINUNITS=1 MAXUNITS=50)**

**/CRITERIA TRAINING=BATCH OPTIMIZATION=SCALEDCONJUGATE LAMBDAINITIAL=0.0000005**

**SIGMAINITIAL=0.00005 INTERVALCENTER=0 INTERVALOFFSET=0.5 MEMSIZE=1000**

**/PRINT CPS NETWORKINFO SUMMARY CLASSIFICATION SOLUTION IMPORTANCE**

**/PLOT NETWORK ROC GAIN LIFT PREDICTED**

**/STOPPINGRULES ERRORSTEPS= 1 (DATA=AUTO) TRAININGTIMER=ON (MAXTIME=15) MAXEPOCHS=AUTO**

**ERRORCHANGE=1.0E-4 ERRORRATIO=0.001**

**/MISSING USERMISSING=EXCLUDE .**

**SET SEED 123.**

*** Decision Tree.**

**TREE binaryclass [n] BY Age [s] BMI [s] Abd_enl [n] percentBCat [s] AreaBC [s]**

**/TREE DISPLAY=TOPDOWN NODES=BOTH BRANCHSTATISTICS=YES NODEDEFS=YES SCALE=AUTO**

**/DEPCATEGORIES USEVALUES=[.00 1.00] TARGET=[1.00]**

**/PRINT MODELSUMMARY IMPORTANCE SURROGATES CLASSIFICATION RISK CATEGORYSPECS TREETABLE**

**/GAIN CATEGORYTABLE=YES TYPE=[NODE] SORT=DESCENDING CUMULATIVE=YES**

**/PLOT IMPORTANCE GAIN INDEX RESPONSE INCREMENT=10**

**/RULES NODES=TERMINAL SYNTAX=INTERNAL TYPE=SCORING SURROGATES=INCLUDE**

**/METHOD TYPE=CRT MAXSURROGATES=AUTO PRUNE=NONE**

**/GROWTHLIMIT MAXDEPTH=4 MINPARENTSIZE=4 MINCHILDSIZE=4**

**/VALIDATION TYPE=CROSSVALIDATION(10) OUTPUT=TESTSAMPLE**

**/CRT IMPURITY=GINI MINIMPROVEMENT=0.0001**

**/COSTS EQUAL**

**/PRIORS FROMDATA ADJUST=NO**

**/MISSING NOMINALMISSING=MISSING.**

**Model 3**

**SET SEED 123.**

***Multilayer Perceptron Network.**

**MLP binaryclass2 (MLEVEL=N) BY Vag_Bleed WITH percentBCat BMI**

**/RESCALE COVARIATE=STANDARDIZED**

**/PARTITION TRAINING=7 TESTING=3 HOLDOUT=0**

**/ARCHITECTURE AUTOMATIC=YES (MINUNITS=1 MAXUNITS=50)**

**/CRITERIA TRAINING=BATCH OPTIMIZATION=SCALEDCONJUGATE LAMBDAINITIAL=0.0000005**

**SIGMAINITIAL=0.00005 INTERVALCENTER=0 INTERVALOFFSET=0.5 MEMSIZE=1000**

**/PRINT CPS NETWORKINFO SUMMARY CLASSIFICATION SOLUTION IMPORTANCE**

**/PLOT NETWORK ROC GAIN LIFT PREDICTED**

**/STOPPINGRULES ERRORSTEPS= 1 (DATA=AUTO) TRAININGTIMER=ON (MAXTIME=15) MAXEPOCHS=AUTO**

**ERRORCHANGE=1.0E-4 ERRORRATIO=0.001**

**/MISSING USERMISSING=EXCLUDE .**

**Model 4**

***Multilayer Perceptron Network.**

**MLP binaryclass2 (MLEVEL=N) BY Vag_Bleed WITH HscoreBC**

**/RESCALE COVARIATE=STANDARDIZED**

**/PARTITION VARIABLE=Data**

**/ARCHITECTURE AUTOMATIC=YES (MINUNITS=1 MAXUNITS=50)**

**/CRITERIA TRAINING=BATCH OPTIMIZATION=SCALEDCONJUGATE LAMBDAINITIAL=0.0000005**

**SIGMAINITIAL=0.00005 INTERVALCENTER=0 INTERVALOFFSET=0.5 MEMSIZE=1000**

**/PRINT CPS NETWORKINFO SUMMARY CLASSIFICATION SOLUTION IMPORTANCE**

**/PLOT NETWORK ROC GAIN LIFT PREDICTED**

**/STOPPINGRULES ERRORSTEPS= 1 (DATA=AUTO) TRAININGTIMER=ON (MAXTIME=15) MAXEPOCHS=AUTO**

**ERRORCHANGE=1.0E-4 ERRORRATIO=0.001**

**/MISSING USERMISSING=EXCLUDE .**

***Non-Atypical Vs Other Stage***

**Model 3**

*** Decision Tree.**

**TREE binaryclass2 [n] BY BMI [s] Vag_Bleed [n] percentBCat [s]**

**/TREE DISPLAY=TOPDOWN NODES=BOTH BRANCHSTATISTICS=YES NODEDEFS=YES SCALE=AUTO**

**/DEPCATEGORIES USEVALUES=[.00 1.00]**

**/PRINT MODELSUMMARY IMPORTANCE SURROGATES CLASSIFICATION RISK CATEGORYSPECS TREETABLE**

**/PLOT IMPORTANCE**

**/RULES NODES=TERMINAL SYNTAX=INTERNAL TYPE=SCORING SURROGATES=INCLUDE**

**/METHOD TYPE=CRT MAXSURROGATES=AUTO PRUNE=NONE**

**/GROWTHLIMIT MAXDEPTH=4 MINPARENTSIZE=4 MINCHILDSIZE=4**

**/VALIDATION TYPE=CROSSVALIDATION(10) OUTPUT=TESTSAMPLE**

**/CRT IMPURITY=GINI MINIMPROVEMENT=0.0001**

**/COSTS EQUAL**

**/PRIORS FROMDATA ADJUST=NO**

**/MISSING NOMINALMISSING=MISSING.**

**Model 4**

*** Decision Tree.**

**TREE binaryclass2 [n] BY Vag_Bleed [n] HscoreBC [s]**

**/TREE DISPLAY=TOPDOWN NODES=BOTH BRANCHSTATISTICS=YES NODEDEFS=YES SCALE=AUTO**

**/DEPCATEGORIES USEVALUES=[.00 1.00]**

**/PRINT MODELSUMMARY IMPORTANCE SURROGATES CLASSIFICATION RISK CATEGORYSPECS TREETABLE**

**/PLOT IMPORTANCE**

**/RULES NODES=TERMINAL SYNTAX=INTERNAL TYPE=SCORING SURROGATES=INCLUDE**

**/METHOD TYPE=CRT MAXSURROGATES=AUTO PRUNE=NONE**

**/GROWTHLIMIT MAXDEPTH=4 MINPARENTSIZE=5 MINCHILDSIZE=5**

**/VALIDATION TYPE=CROSSVALIDATION(10) OUTPUT=TESTSAMPLE**

**/CRT IMPURITY=GINI MINIMPROVEMENT=0.0001**

**/COSTS EQUAL**

**/PRIORS FROMDATA ADJUST=NO**

**/MISSING NOMINALMISSING=MISSING.**
