# Supplementary material for: Risk-stratification machine learning model using demographic factors, gynaecological symptoms and β-catenin for endometrial hyperplasia and carcinoma: a cross-sectional study
Source: BMC Womens Health. 2023 Nov 27;23:627. doi: 10.1186/s12905-023-02790-6 (PMC10680196; doi:10.1186/s12905-023-02790-6)
Supplement: Supplementary file 2 — Additional file 2: Supplementary Table 1. Characteristics of participants based on carcinoma and non-carcinoma findings. Supplementary Table 2. Characteristics of participants based on non-atypical and later stages findings. Supplementary Table 3. Generalized ordinal logistic regression of three pathology classes considering the h-score of β-catenin. Supplementary Table 4. Model structure of artificial neural network in predicting carcinoma using % of beta-catenin. Supplementary Table 5. Model structure of artificial neural network in predicting carcinoma using h-score of beta-catenin. Supplementary Table 6. Model structure of artificial neural network in predicting non-atypical finding using % of beta-catenin. Supplementary Table 7. Model structure of artificial neural network in predicting non-atypical finding using h-score of beta-catenin. Supplementary Table 8. Model structure of decision tree in predicting non-atypical finding using % beta-catenin. Supplementary Table 9. Model structure of decision tree in predicting non-atypical finding using h-score of beta-catenin. Supplementary Table 10. Summary of model performance. Supplementary Table 11. Additional analysis. [file 12905_2023_2790_MOESM2_ESM.docx]

Contents

[SUPPLEMENTARY TABLE 1. CHARACTERISTICS OF PARTICIPANTS BASED ON CARCINOMA AND NON-CARCINOMA FINDINGS 2](#_Toc141726617)

[SUPPLEMENTARY TABLE 2. CHARACTERISTICS OF PARTICIPANTS BASED ON NON-ATYPICAL AND LATER STAGES FINDINGS 3](#_Toc141726618)

[SUPPLEMENTARY TABLE 3. GENERALIZED ORDINAL LOGISTIC REGRESSION OF THREE PATHOLOGY CLASSES CONSIDERING THE H-SCORE OF Β-CATENIN 4](#_Toc141726619)

[SUPPLEMENTARY TABLE 4. MODEL STRUCTURE OF ARTIFICIAL NEURAL NETWORK IN PREDICTING CARCINOMA USING % OF BETA-CATENIN 5](#_Toc141726620)

[SUPPLEMENTARY TABLE 5. MODEL STRUCTURE OF ARTIFICIAL NEURAL NETWORK IN PREDICTING CARCINOMA USING H-SCORE OF BETA-CATENIN 8](#_Toc141726621)

[SUPPLEMENTARY TABLE 6. MODEL STRUCTURE OF ARTIFICIAL NEURAL NETWORK IN PREDICTING NON-ATYPICAL FINDING USING % OF BETA-CATENIN 11](#_Toc141726622)

[SUPPLEMENTARY TABLE 7. MODEL STRUCTURE OF ARTIFICIAL NEURAL NETWORK IN PREDICTING NON-ATYPICAL FINDING USING H-SCORE OF BETA-CATENIN 14](#_Toc141726623)

[SUPPLEMENTARY TABLE 8. MODEL STRUCTURE OF DECISION TREE IN PREDICTING NON-ATYPICAL FINDING USING % BETA-CATENIN 17](#_Toc141726624)

[SUPPLEMENTARY TABLE 9. MODEL STRUCTURE OF DECISION TREE IN PREDICTING NON-ATYPICAL FINDING USING H-SCORE OF BETA-CATENIN 19](#_Toc141726625)

[SUPPLEMENTARY TABLE 10. SUMMARY OF MODEL PERFORMANCE 21](#_Toc141726626)

[SUPPLEMENTARY TABLE 11. ADDITIONAL ANALYSIS 22](#_Toc141726627)

| **Variables** | | **Non-carcinoma (n=59)** | **Endometrial Carcinoma (31)** | **p-value** |
| --- | --- | --- | --- | --- |
| Age* | | 42.85 ± 8.57 | 49.29 ± 8.59 | <0.001 |
| Body Mass Index | | 27.19 ± 5.21 | 27.57 ± 5.33 | 0.552 |
| Parity Episode^ | | 2 (IQR 0-5) | 3 (IQR 1-4) | 0.052 |
| Abortion Episode^ | | 0 (IQR 0-1) | 1 (IQR 0-2) | 0.190 |
| Signs, Symptoms and Previous intervention | |  | | |
|  | Vaginal Bleeding | 30 (50.8%) | 15 (48.4%) | 0.824 |
|  | Abdominal Enlargement | 6 (10.2%) | 13 (41.9%) | <0.001 |
|  | Mennorrhagia# | 12 (20.3%) | 1 (3.2%) | 0.030 |
|  | Mennometroraghia | 13 (22.0%) | 6 (19.3%) | 0.767 |
|  | Uterus Bleeding | 34 (57.6%) | 23 (74.2%) | 0.121 |
|  | Abdominal Pain# | 3 (5.1%) | 4 (12.9%) | 0.228 |
|  | Hormon Replacement Therapy# | 3 (5.1%) | 2 (6.4%) | 1.000 |
|  | Previous Curretage# | 1 (1.7%) | 1 (3.2%) | 1.000 |
| Referral Diagnosis | |  |  |  |
|  | Abnormal Uterus Bleeding | 26 (44.1%) | 1 (3.2%) |  |
|  | Endometrial Hyperplasia | 24 (40.7%) | 4 (12.9%) |  |
|  | Ovarian Carcinoma | 2 (3.4%) | 1 (3.2%) |  |
|  | Adenomyosis | 1 (1.7%) | 0 (0%) |  |
|  | Endometrial Cysts | 2 (3.4%) | 0 (0%) |  |
|  | Myoma Uteri | 9 (15.3%) | 2 (6.4%) |  |
|  | Cervical Polyp | 1 (1.7%) | 0 (0%) |  |
|  | Endometrial Polyp | 1 (1.7%) | 0 (0%) |  |
|  | Endometrial Carcinoma | 1 (1.7%) | 24 (77.4%) |  |
|  | Cyst Torsion | 0 (0%) | 1 (3.2%) |  |
| β-Catenin | Intensity | 2.76 ± 0.43 | 2.77 ± 0.42 | 0.903 |
|  | Percentage | 58.81 ± 22.52 | 63.87 ± 23.48 | 0.263 |
|  | Area Percentage | 2.58 ± 0.53 | 2.65 ± 0.61 | 0.380 |
|  | H-Score | 168.31 ± 75.23 | 184.51 ± 68.30 | 0.383 |

## SUPPLEMENTARY TABLE 1. CHARACTERISTICS OF PARTICIPANTS BASED ON CARCINOMA AND NON-CARCINOMA FINDINGS

## SUPPLEMENTARY TABLE 2. CHARACTERISTICS OF PARTICIPANTS BASED ON NON-ATYPICAL AND LATER STAGES FINDINGS

| **Variables** | | **Non-Atypical (n=32)** | **Atypical + Carcinoma (n=58)** | **p-value** |
| --- | --- | --- | --- | --- |
| Age* | | 44.97 ± 6.15 | 45.12 ± 10.39 | 0.931 |
| Body Mass Index | | 26.12 ± 5.37 | 27.99 ± 5.06 | 0.034 |
| Parity Episode^ | | 2 (IQR 0-5) | 2 (IQR 0-5) | 0.777 |
| Abortion Episode^ | | 0 (IQR 0-1) | 0 (IQR 0-2) | 0.168 |
| Signs, Symptoms and Previous Intervention | |  | | |
|  | Vaginal Bleeding | 12 (37.5%) | 33 (56.9%) | 0.078 |
|  | Abdominal Enlargement | 5 (15.6%) | 14 (24.1%) | 0.343 |
|  | Mennorrhagia# | 6 (18.8%) | 7 (12.1%) | 0.532 |
|  | Mennometroraghia | 5 (15.6%) | 14 (24.1%) | 0.343 |
|  | Uterus Bleeding | 20 (62.5%) | 37 (63.8%) | 0.903 |
|  | Abdominal Pain# | 1 (3.1%) | 6 (10.3%) | 0.414 |
|  | Hormone Replacement Therapy# | 2 (6.2%) | 3 (5.2%) | 1.000 |
|  | Previous Curretage# | 0 (0%) | 2 (3.4%) | 0.537 |
| Referral Diagnosis | |  |  |  |
|  | Abnormal Uterus Bleeding | 16 (50%) | 11 (18.9%) |  |
|  | Endometrial Hyperplasia | 13 (40.1%) | 15 (25.9%) |  |
|  | Ovarian Carcinoma | 1 (3.1%) | 2 (3.4%) |  |
|  | Adenomyosis | 0 (0%) | 1 (1.7%) |  |
|  | Endometrial Cysts | 0 (0%) | 2 (3.4%) |  |
|  | Myoma Uteri | 6 (18.8%) | 5 (8.6%) |  |
|  | Cervical Polyp | 0 (0%) | 1 (1.7%) |  |
|  | Endometrial Polyp | 0 (0%) | 1 (1.7%) |  |
|  | Endometrial Carcinoma | 0 (0%) | 25 (43.1%) |  |
|  | Cyst Torsion | 0 (0%) | 1 (1.7%) |  |
| β-Catenin | Intensity | 2.59 ± 0.49 | 2.86 ± 0.35 | 0.004 |
|  | Percentage | 50.94 ± 22.63 | 65.86 ± 21.36 | 0.002 |
|  | Area Percentage | 2.41 ± 0.49 | 2.71 ± 0.56 | 0.003 |
|  | H-Score | 138.13 ± 75.79 | 193.62 ± 63.79 | 0.001 |

## SUPPLEMENTARY TABLE 3. GENERALIZED ORDINAL LOGISTIC REGRESSION OF THREE PATHOLOGY CLASSES CONSIDERING THE H-SCORE OF Β-CATENIN

| **Parameter** | | **B** | **Std. Error** | **Sig.** | **Exp(B)** | **95% Wald Confidence Interval for Exp(B)** | |
| --- | --- | --- | --- | --- | --- | --- | --- |
|  |  |  |  |  |  | **Lower** | **Upper** |
| Threshold | Atypical | 5.014 | 2.0487 | 0.014 | 150.551 | 2.715 | 8346.986 |
|  | Carcinoma | 6.525 | 2.1043 | 0.002 | 682.236 | 11.034 | 42184.698 |
| Vaginal Bleeding | | 0.549 | 0.4229 | 0.194 | 1.732 | 0.756 | 3.966 |
| Abdominal Enlargement | | 1.858 | 0.6658 | 0.005 | 6.408 | 1.738 | 23.632 |
| Menorrhagia | | -0.131 | 0.5776 | 0.821 | 0.878 | 0.283 | 2.723 |
| Age | | 0.037 | 0.0305 | 0.223 | 1.038 | 0.978 | 1.102 |
| Parity | | -0.049 | 0.2030 | 0.810 | 0.952 | 0.640 | 1.418 |
| Body Mass Index | | 0.074 | 0.0448 | 0.098 | 1.077 | 0.987 | 1.176 |
| H-Score β-Catenin | | 0.009 | 0.0030 | 0.004 | 1.009 | 1.003 | 1.015 |

## SUPPLEMENTARY TABLE 4. MODEL STRUCTURE OF ARTIFICIAL NEURAL NETWORK IN PREDICTING CARCINOMA USING % OF BETA-CATENIN

| **Case Processing Summary** | | | |
| --- | --- | --- | --- |
|  | | N | Per cent |
| Sample | Training | 63 | 70.0% |
|  | Testing | 27 | 30.0% |
| Valid | | 90 | 100.0% |
| Excluded | | 0 |  |
| Total | | 90 |  |

| **Network Information** | | | |
| --- | --- | --- | --- |
| Input Layer | Factors | 1 | Uterus Bleeding |
|  |  | 2 | Abdominal Enlargement |
|  | Covariates | 1 | Age |
|  |  | 2 | % Beta-Catenin |
|  |  | 3 | BMI |
|  | Number of Units^a^ | | 7 |
|  | Rescaling Method for Covariates | | Standardized |
| Hidden Layer(s) | Number of Hidden Layers | | 1 |
|  | Number of Units in Hidden Layer 1^a^ | | 4 |
|  | Activation Function | | Hyperbolic tangent |
| Output Layer | Dependent Variables | 1 | Carcinoma Vs Non Carcinoma |
|  | Number of Units | | 2 |
|  | Activation Function | | Softmax |
|  | Error Function | | Cross-entropy |
| a. Excluding the bias unit | | | |

MODEL STRUCTURE


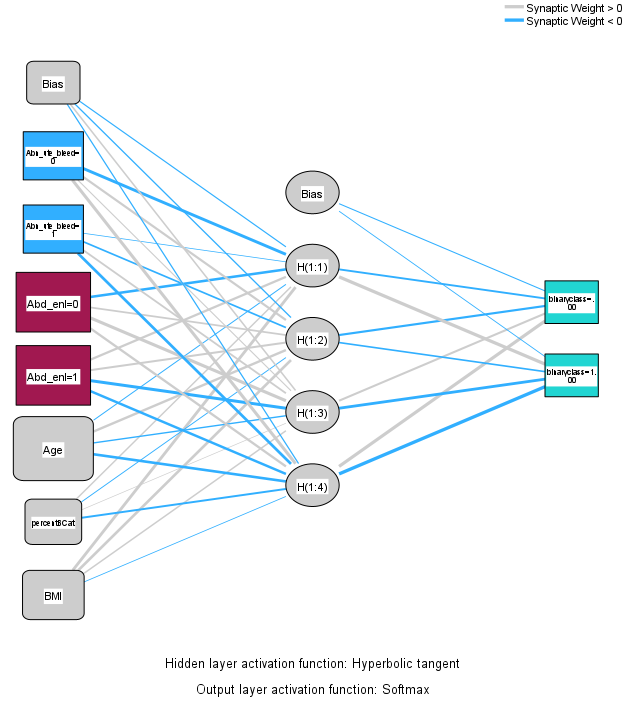


| **Model Summary** | | | | | |  |  |
| --- | --- | --- | --- | --- | --- | --- | --- |
| Training | | Cross Entropy Error | | 27.628 | |  |  |
|  |  | Percent Incorrect Predictions | | 23.8% | |  |  |
|  |  | Stopping Rule Used | | 1 consecutive step(s) with no decrease in error^a^ | |  |  |
|  |  | Training Time | | 0:00:00.03 | |  |  |
| Testing | | Cross Entropy Error | | 13.340 | |  |  |
|  |  | Percent Incorrect Predictions | | 18.5% | |  |  |
| Dependent Variable: Carcinoma Vs Non Carcinoma | | | | | |  |  |
| a. Error computations are based on the testing sample. | | | | | |  |  |
| **Classification** | | | | | | | |
| Sample | Observed | | Predicted | | | | |
|  |  |  | Non Carcinoma | | Carcinoma | | Percent Correct |
| Training | Non Carcinoma | | 38 | | 4 | | 90.5% |
|  | Carcinoma | | 11 | | 10 | | 47.6% |
|  | Overall Percent | | 77.8% | | 22.2% | | 76.2% |
| Testing | Non Carcinoma | | 15 | | 2 | | 88.2% |
|  | Carcinoma | | 3 | | 7 | | 70.0% |
|  | Overall Percent | | 66.7% | | 33.3% | | 81.5% |
| Dependent Variable: Carcinoma Vs Non Carcinoma | | | | | | | |

| **Area Under the Curve** | | |
| --- | --- | --- |
|  | | Area |
| Carcinoma Vs Non Carcinoma | Non Carcinoma | .841 |
|  | Carcinoma | .841 |

| **Independent Variable Importance** | | |
| --- | --- | --- |
|  | Importance | Normalized Importance |
| Uterus Bleeding | .168 | 62.8% |
| Abdominal Enlargement | .239 | 89.5% |
| Age | .267 | 100.0% |
| % Beta Catenin | .150 | 56.2% |
| BMI | .175 | 65.5% |

## SUPPLEMENTARY TABLE 5. MODEL STRUCTURE OF ARTIFICIAL NEURAL NETWORK IN PREDICTING CARCINOMA USING H-SCORE OF BETA-CATENIN

| **Case Processing Summary** | | | |
| --- | --- | --- | --- |
|  | | N | Percent |
| Sample | Training | 63 | 70.0% |
|  | Testing | 27 | 30.0% |
| Valid | | 90 | 100.0% |
| Excluded | | 0 |  |
| Total | | 90 |  |

| **Network Information** | | | |
| --- | --- | --- | --- |
| Input Layer | Factors | 1 | Uterus Bleeding |
|  |  | 2 | Abdominal Enlargement |
|  | Covariates | 1 | Age |
|  |  | 2 | BMI |
|  |  | 3 | H-Score Beta Catenin |
|  | Number of Units^a^ | | 7 |
|  | Rescaling Method for Covariates | | Standardized |
| Hidden Layer(s) | Number of Hidden Layers | | 1 |
|  | Number of Units in Hidden Layer 1^a^ | | 4 |
|  | Activation Function | | Hyperbolic tangent |
| Output Layer | Dependent Variables | 1 | Carcinoma Vs Non Carcinoma |
|  | Number of Units | | 2 |
|  | Activation Function | | Softmax |
|  | Error Function | | Cross-entropy |
| a. Excluding the bias unit | | | |

MODEL STRUCTURE


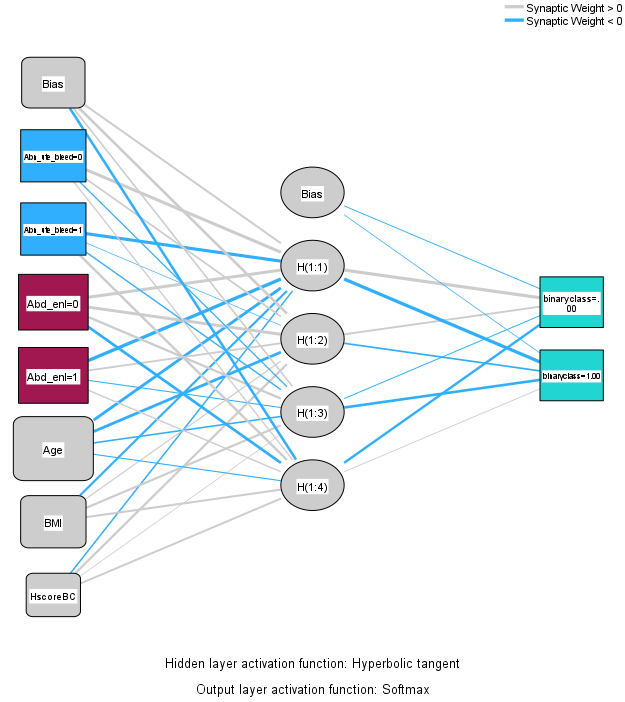


| **Model Summary** | | | | | |  |  |
| --- | --- | --- | --- | --- | --- | --- | --- |
| Training | | Cross Entropy Error | | 28.449 | |  |  |
|  |  | Percent Incorrect Predictions | | 19.0% | |  |  |
|  |  | Stopping Rule Used | | 1 consecutive step(s) with no decrease in error^a^ | |  |  |
|  |  | Training Time | | 0:00:00.02 | |  |  |
| Testing | | Cross Entropy Error | | 13.231 | |  |  |
|  |  | Percent Incorrect Predictions | | 18.5% | |  |  |
| Dependent Variable: Carcinoma Vs Non Carcinoma | | | | | |  |  |
| a. Error computations are based on the testing sample. | | | | | |  |  |
| **Classification** | | | | | | | |
| Sample | Observed | | Predicted | | | | |
|  |  |  | Non Carcinoma | | Carcinoma | | Percent Correct |
| Training | Non Carcinoma | | 37 | | 5 | | 88.1% |
|  | Carcinoma | | 7 | | 14 | | 66.7% |
|  | Overall Percent | | 69.8% | | 30.2% | | 81.0% |
| Testing | Non Carcinoma | | 16 | | 1 | | 94.1% |
|  | Carcinoma | | 4 | | 6 | | 60.0% |
|  | Overall Percent | | 74.1% | | 25.9% | | 81.5% |
| Dependent Variable: Carcinoma Vs Non Carcinoma | | | | | | | |

| **Area Under the Curve** | | |
| --- | --- | --- |
|  | | Area |
| Carcinoma Vs Non Carcinoma | Non Carcinoma | .837 |
|  | Carcinoma | .837 |

| **Independent Variable Importance** | | |
| --- | --- | --- |
|  | Importance | Normalized Importance |
| Uterus Bleeding | .184 | 55.8% |
| Abdominal Enlargement | .227 | 68.8% |
| Age | .329 | 100.0% |
| BMI | .185 | 56.1% |
| H-Score Beta Catenin | .075 | 22.8% |

## SUPPLEMENTARY TABLE 6. MODEL STRUCTURE OF ARTIFICIAL NEURAL NETWORK IN PREDICTING NON-ATYPICAL FINDING USING % OF BETA-CATENIN

| **Case Processing Summary** | | | |
| --- | --- | --- | --- |
|  | | N | Percent |
| Sample | Training | 60 | 66.7% |
|  | Testing | 30 | 33.3% |
| Valid | | 90 | 100.0% |
| Excluded | | 0 |  |
| Total | | 90 |  |

| **Network Information** | | | |
| --- | --- | --- | --- |
| Input Layer | Factors | 1 | Vaginal Bleeding |
|  | Covariates | 1 | % Beta Catenin |
|  |  | 2 | BMI |
|  | Number of Units^a^ | | 4 |
|  | Rescaling Method for Covariates | | Standardized |
| Hidden Layer(s) | Number of Hidden Layers | | 1 |
|  | Number of Units in Hidden Layer 1^a^ | | 5 |
|  | Activation Function | | Hyperbolic tangent |
| Output Layer | Dependent Variables | 1 | Non Atypical Vs Atypical + Carcinoma |
|  | Number of Units | | 2 |
|  | Activation Function | | Softmax |
|  | Error Function | | Cross-entropy |
| a. Excluding the bias unit | | | |

Model Structure


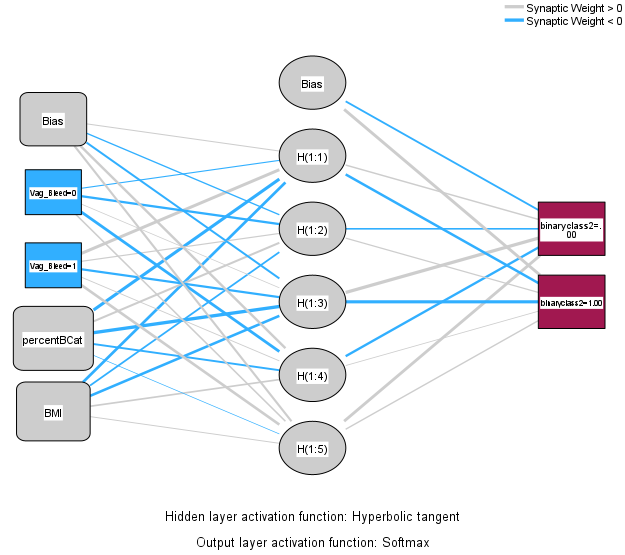


| **Model Summary** | | | | | |  |  |
| --- | --- | --- | --- | --- | --- | --- | --- |
| Training | | Cross Entropy Error | | 32.621 | |  |  |
|  |  | Percent Incorrect Predictions | | 23.3% | |  |  |
|  |  | Stopping Rule Used | | 1 consecutive step(s) with no decrease in error^a^ | |  |  |
|  |  | Training Time | | 0:00:00.02 | |  |  |
| Testing | | Cross Entropy Error | | 19.184 | |  |  |
|  |  | Percent Incorrect Predictions | | 30.0% | |  |  |
| Dependent Variable: Non Atypical Vs Atypical + Carcinoma | | | | | |  |  |
| a. Error computations are based on the testing sample. | | | | | |  |  |
| **Classification** | | | | | | | |
| Sample | Observed | | Predicted | | | | |
|  |  |  | Non-Atypical | | Atypical + Carcinoma | | Percent Correct |
| Training | Non-Atypical | | 8 | | 9 | | 47.1% |
|  | Atypical + Carcinoma | | 5 | | 38 | | 88.4% |
|  | Overall Percent | | 21.7% | | 78.3% | | 76.7% |
| Testing | Non-Atypical | | 9 | | 6 | | 60.0% |
|  | Atypical + Carcinoma | | 3 | | 12 | | 80.0% |
|  | Overall Percent | | 40.0% | | 60.0% | | 70.0% |
| Dependent Variable: Non-Atypical Vs Atypical + Carcinoma | | | | | | | |

| **Area Under the Curve** | | |
| --- | --- | --- |
|  | | Area |
| Non-Atypical Versus  Atypical + Carcinoma | Non-Atypical | .714 |
|  | Atypical + Carcinoma | .714 |

| **Independent Variable Importance** | | |
| --- | --- | --- |
|  | Importance | Normalized Importance |
| Vaginal Bleeding | .055 | 10.3% |
| % Beta Catenin | .536 | 100.0% |
| BMI | .408 | 76.1% |

## SUPPLEMENTARY TABLE 7. MODEL STRUCTURE OF ARTIFICIAL NEURAL NETWORK IN PREDICTING NON-ATYPICAL FINDING USING H-SCORE OF BETA-CATENIN

| **Case Processing Summary** | | | |
| --- | --- | --- | --- |
|  | | N | Percent |
| Sample | Training | 63 | 70.0% |
|  | Testing | 27 | 30.0% |
| Valid | | 90 | 100.0% |
| Excluded | | 0 |  |
| Total | | 90 |  |

| **Network Information** | | | |
| --- | --- | --- | --- |
| Input Layer | Factors | 1 | Vaginal Bleeding |
|  | Covariates | 1 | H-Score Beta Catenin |
|  | Number of Units^a^ | | 3 |
|  | Rescaling Method for Covariates | | Standardized |
| Hidden Layer(s) | Number of Hidden Layers | | 1 |
|  | Number of Units in Hidden Layer 1^a^ | | 4 |
|  | Activation Function | | Hyperbolic tangent |
| Output Layer | Dependent Variables | 1 | Non-Atypical Vs Atypical + Carcinoma |
|  | Number of Units | | 2 |
|  | Activation Function | | Softmax |
|  | Error Function | | Cross-entropy |
| a. Excluding the bias unit | | | |

MODEL STRUCTURE


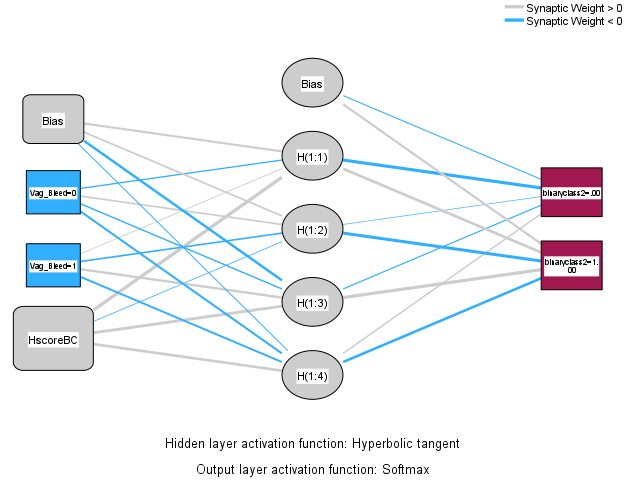


| **Model Summary** | | | | | |  |  |
| --- | --- | --- | --- | --- | --- | --- | --- |
| Training | | Cross Entropy Error | | 33.176 | |  |  |
|  |  | Percent Incorrect Predictions | | 28.6% | |  |  |
|  |  | Stopping Rule Used | | 1 consecutive step(s) with no decrease in error^a^ | |  |  |
|  |  | Training Time | | 0:00:00.02 | |  |  |
| Testing | | Cross Entropy Error | | 17.408 | |  |  |
|  |  | Percent Incorrect Predictions | | 29.6% | |  |  |
| Dependent Variable: Non-Atypical Vs Atypical + Carcinoma | | | | | |  |  |
| a. Error computations are based on the testing sample. | | | | | |  |  |
| **Classification** | | | | | | | |
| Sample | Observed | | Predicted | | | | |
|  |  |  | Non-Atypical | | Atypical + Carcinoma | | Percent Correct |
| Training | Non-Atypical | | 11 | | 10 | | 52.4% |
|  | Atypical + Carcinoma | | 8 | | 34 | | 81.0% |
|  | Overall Percent | | 30.2% | | 69.8% | | 71.4% |
| Testing | Non-Atypical | | 5 | | 6 | | 45.5% |
|  | Atypical + Carcinoma | | 2 | | 14 | | 87.5% |
|  | Overall Percent | | 25.9% | | 74.1% | | 70.4% |
| Dependent Variable: Non-Atypical Vs Atypical + Carcinoma | | | | | | | |

| **Area Under the Curve** | | | | | |
| --- | --- | --- | --- | --- | --- |
|  | | | | Area | |
| Non-Atypical Vs Atypical + Carcinoma | | Non-Atypical | | .741 | |
|  |  | Atypical + Carcinoma | | .741 | |
| **Independent Variable Importance** | | | | |  |
|  | Importance | | Normalized Importance | |  |
| Vaginal Bleeding | .240 | | 31.6% | |  |
| H-Score Beta Catenin | .760 | | 100.0% | |  |

## SUPPLEMENTARY TABLE 8. MODEL STRUCTURE OF DECISION TREE IN PREDICTING NON-ATYPICAL FINDING USING % BETA-CATENIN

| **Model Summary** | | |
| --- | --- | --- |
| Specifications | Growing Method | CRT |
|  | Dependent Variable | Non Atypical Vs Atypical + Carcinoma |
|  | Independent Variables | BMI, Vaginal Bleeding, % Beta-Catenin |
|  | Validation | Cross Validation |
|  | Maximum Tree Depth | 4 |
|  | Minimum Cases in Parent Node | 4 |
|  | Minimum Cases in Child Node | 4 |
| Results | Independent Variables Included | BMI, Vaginal Bleeding, % Beta-Catenin |
|  | Number of Nodes | 5 |
|  | Number of Terminal Nodes | 3 |
|  | Depth | 2 |

| **Classification** | | | |
| --- | --- | --- | --- |
| Observed | Predicted | | |
|  | Non Atypical | Atypical + Carcinoma | Percent Correct |
| Non Atypical | 13 | 19 | 40.6% |
| Atypical + Carcinoma | 3 | 55 | 94.8% |
| Overall Percentage | 17.8% | 82.2% | 75.6% |
| Growing Method: CRT  Dependent Variable: Non Atypical Vs Atypical + Carcinoma | | | |

| **Independent Variable Importance** | | |
| --- | --- | --- |
| Independent Variable | Importance | Normalized Importance |
| % Beta Catenin | .080 | 100.0% |
| BMI | .040 | 50.3% |
| Vaginal Bleeding | .013 | 16.4% |
| Growing Method: CRT  Dependent Variable: Non Atypical Vs Atypical + Carcinoma | | |


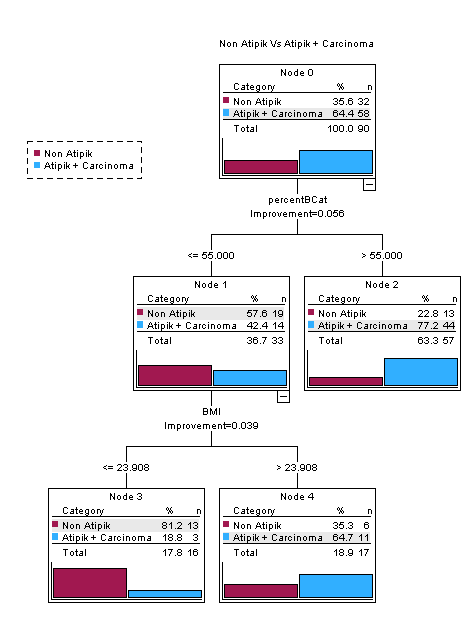


## SUPPLEMENTARY TABLE 9. MODEL STRUCTURE OF DECISION TREE IN PREDICTING NON-ATYPICAL FINDING USING H-SCORE OF BETA-CATENIN

| **Model Summary** | | |
| --- | --- | --- |
| Specifications | Growing Method | CRT |
|  | Dependent Variable | Non Atypical Vs Atypical + Carcinoma |
|  | Independent Variables | Vaginal Bleeding, H-Score Beta Catenin |
|  | Validation | Cross Validation |
|  | Maximum Tree Depth | 4 |
|  | Minimum Cases in Parent Node | 5 |
|  | Minimum Cases in Child Node | 5 |
| Results | Independent Variables Included | H-Score Beta Catenin, Vaginal Bleeding |
|  | Number of Nodes | 5 |
|  | Number of Terminal Nodes | 3 |
|  | Depth | 2 |

| **Classification** | | | |
| --- | --- | --- | --- |
| Observed | Predicted | | |
|  | Non Atypical | Atypical + Carcinoma | Percent Correct |
| Non Atypical | 16 | 16 | 50.0% |
| Atypical + Carcinoma | 6 | 52 | 89.7% |
| Overall Percentage | 24.4% | 75.6% | 75.6% |
| Growing Method: CRT  Dependent Variable: Non Atypical Vs Atypical + Carcinoma | | | |

| **Independent Variable Importance** | | |
| --- | --- | --- |
| Independent Variable | Importance | Normalized Importance |
| H-Score Beta Catenin | .089 | 100.0% |
| Vaginal Bleeding | .003 | 3.2% |
| Growing Method: CRT  Dependent Variable: Non Atypical Vs Atypical + Carcinoma | | |


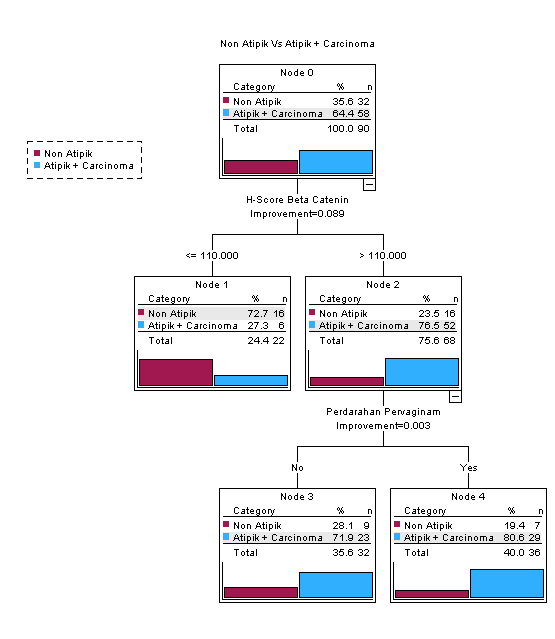


## SUPPLEMENTARY TABLE 10. SUMMARY OF MODEL PERFORMANCE

| **Carcinoma Versus Non-Carcinoma** | **Sensitivity** | **Specificity** | **Accuracy** |
| --- | --- | --- | --- |
| Neural Network Model 1 | 70.0% | 88.2% | 81.5% |
| Neural Network Model 2 | 60.0% | 94.1% | 81.5% |
| **Non-Atypical Versus Atypical + Carcinoma** | **Sensitivity** | **Specificity** | **Accuracy** |
| Neural Network Model 3 | 80.0% | 60.0% | 70.0% |
| Neural Network Model 4 | 87.5% | 45.5% | 70.4% |
| Decision Tree Model 3 | 94.8% | 40.6% | 75.6% |
| Decision Tree Model 4 | 89.7% | 50.0% | 75.6% |

## SUPPLEMENTARY TABLE 11. ADDITIONAL ANALYSIS

**11A. Correlation Continous Data**

| SPEARMAN CORRELATION | | H-Score | Percentage | Intensity |
| --- | --- | --- | --- | --- |
| Age | Correlation Coefficient | 0.025 | 0.011 | -0.004 |
|  | Sig. (2-tailed) | 0.818 | 0.917 | 0.970 |
|  | N | 90 | 90 | 90 |
| BMI | Correlation Coefficient | 0.160 | 0.056 | .251^*^ |
|  | Sig. (2-tailed) | 0.132 | 0.597 | 0.017 |
|  | N | 90 | 90 | 90 |
| Parity | Correlation Coefficient | 0.068 | 0.031 | -0.010 |
|  | Sig. (2-tailed) | 0.527 | 0.772 | 0.926 |
|  | N | 90 | 90 | 90 |
| Abortion | Correlation Coefficient | .254^*^ | 0.179 | .250^*^ |
|  | Sig. (2-tailed) | 0.016 | 0.092 | 0.018 |
|  | N | 90 | 90 | 90 |

| **Hypothesis Test Summary** | | | |
| --- | --- | --- | --- |
|  | **Null Hypothesis** | **Test** | **p-value** |
| 1 | The distribution of Intentitas Beta Catenin is the same across categories of Menorrhagia. | Independent-Samples Mann-Whitney U Test | 0.496 |
| 2 | The distribution of H-Score Beta Catenin is the same across categories of Menorrhagia. | Independent-Samples Mann-Whitney U Test | 0.435 |
| 3 | The distribution of percentage Beta Catenin is the same across categories of Menorrhagia. | Independent-Samples Mann-Whitney U Test | 0.310 |

**11B. Difference of beta-catenin value according to menorrhagia status**
