# Supplementary material for: Risk-stratification machine learning model using demographic factors, gynaecological symptoms and β-catenin for endometrial hyperplasia and carcinoma: a cross-sectional study
Source: BMC Womens Health. 2023 Nov 27;23:627. doi: 10.1186/s12905-023-02790-6 (PMC10680196; doi:10.1186/s12905-023-02790-6)

Contents

[1. Supplementary Figure 1 2](#_Toc141727052)

[2. Supplementary Figure 2. 3](#_Toc141727053)

# 1. Supplementary Figure 1

Violin plots of β-catenin percentage and H-Score according to the three-level of histopathology. The median of %β-catenin and H-Score were higher in Atypical, followed by carcinoma and non-atypical hyperplasia.


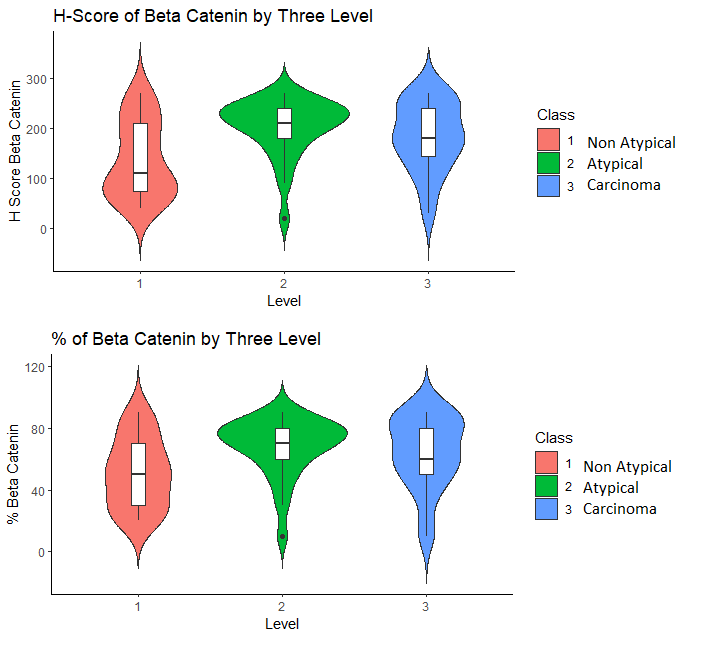


# 2. Supplementary Figure 2.

ROC Curve of β-Catenin and H-Score in Differentiating Carcinoma, and Non-Atypical. Not that all ROC curve intersect the diagonal line, thus conclusion should not be made when considering beta-catenin as a single discriminant factor


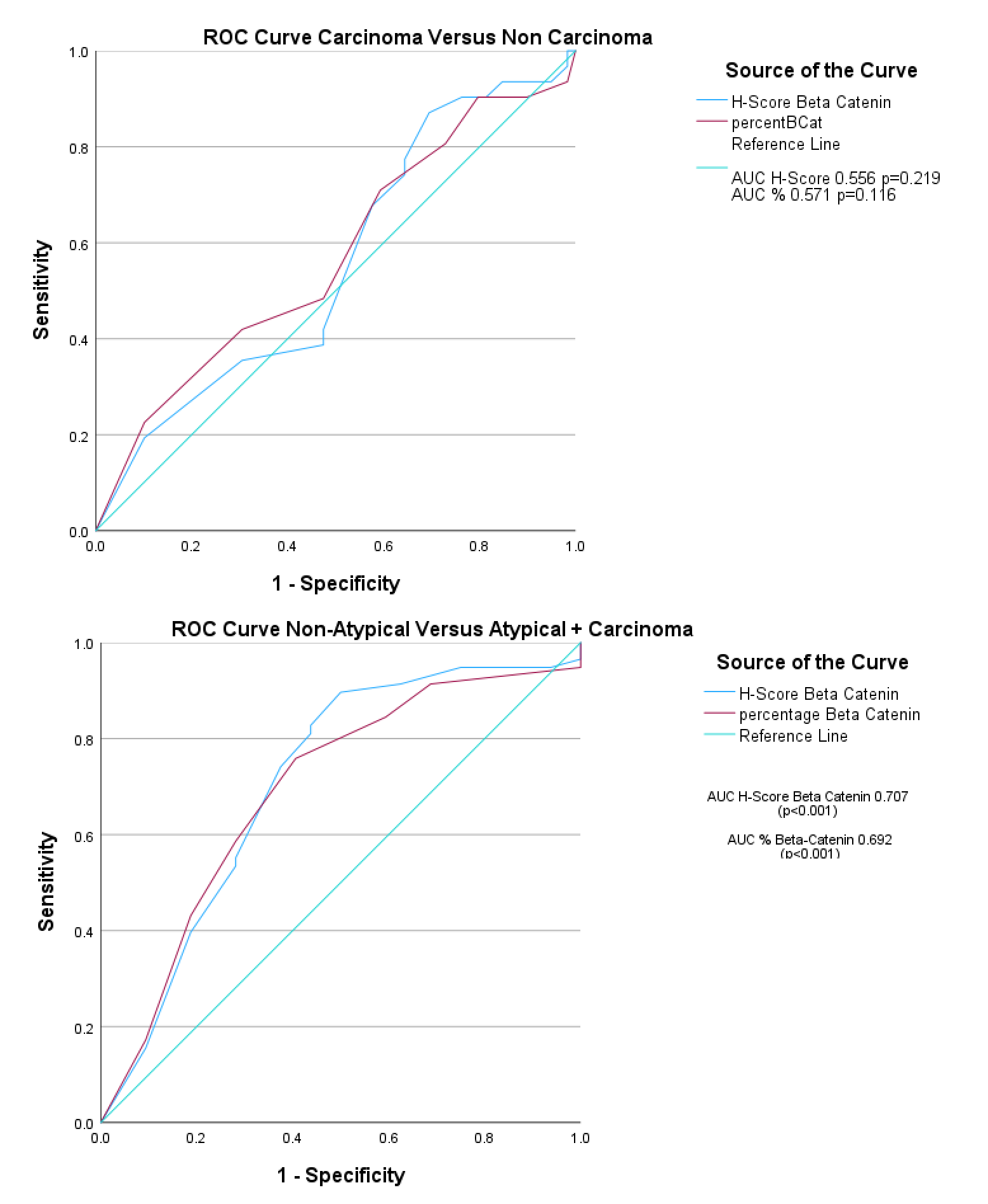

Supplement: Supplementary file 3 — Additional file 3: Supplementary Figure 1. Violin plots of β-catenin percentage and H-Score according to the three-level of histopathology. The median of %β-catenin and H-Score were higher in Atypical, followed by carcinoma and non-atypical hyperplasia. Supplementary Figure 2. ROC Curve of β-Catenin and H-Score in Differentiating Carcinoma, and Non-Atypical. Not that all ROC curve intersect the diagonal line, thus conclusion should not be made when considering beta-catenin as a single discriminant factor. [file 12905_2023_2790_MOESM3_ESM.docx]
